# Monitoring the mass, eigenfrequency, and quality factor of mammalian cells

**DOI:** 10.1038/s41467-024-46056-7

**Published:** 2024-02-26

**Authors:** Sophie Herzog, Gotthold Fläschner, Ilaria Incaviglia, Javier Casares Arias, Aaron Ponti, Nico Strohmeyer, Michele M. Nava, Daniel J. Müller

**Affiliations:** 1 Department of Biosystems Science and Engineering, Eidgenössische Technische Hochschule (ETH) Zurich, Klingelbergstrasse 48, 4056 Basel, Switzerland; 2 Nanosurf AG, Gräubernstrasse 12, 4410 Liestal, Switzerland

**Keywords:** Biomaterials - cells, Nanoscale biophysics, Cytoskeleton, Biological physics

## Abstract

The regulation of mass is essential for the development and homeostasis of cells and multicellular organisms. However, cell mass is also tightly linked to cell mechanical properties, which depend on the time scales at which they are measured and change drastically at the cellular eigenfrequency. So far, it has not been possible to determine cell mass and eigenfrequency together. Here, we introduce microcantilevers oscillating in the Ångström range to monitor both fundamental physical properties of the cell. If the oscillation frequency is far below the cellular eigenfrequency, all cell compartments follow the cantilever motion, and the cell mass measurements are accurate. Yet, if the oscillating frequency approaches or lies above the cellular eigenfrequency, the mechanical response of the cell changes, and not all cellular components can follow the cantilever motions in phase. This energy loss caused by mechanical damping within the cell is described by the quality factor. We use these observations to examine living cells across externally applied mechanical frequency ranges and to measure their total mass, eigenfrequency, and quality factor. The three parameters open the door to better understand the mechanobiology of the cell and stimulate biotechnological and medical innovations.

## Abstract (web summary)

There is increasing interest in measuring the mechanical properties of living cells. Here, the authors develop a method to simultaneously measure the cell mass and two parameters related to its natural oscillation or resonance frequencies.

## Introduction

Measurement of how mammalian cells regulate size, growth, and mechanical properties is of considerable interest for research fields including cell biology, developmental biology, biophysics, tissue engineering, biomaterials, and physiology, and for a better understanding of diseases including cancer, fibrosis, inflammation, and impaired wound healing^1–3^. Cell size and growth can be described via fundamental physical properties, such as mass or volume, which are linked to complex biological processes. For instance, dividing cells drastically change shape to grow daughter cells, while considerably modulating their mechanical properties such as pressure, cortex stiffness, and adhesion^4–9^. Cells also continuously monitor the spatial constraints of the extracellular environment to which they respond mechanically^10–13^. However, despite the enormous importance for cellular fitness and function, the mechanisms of how cells regulate mass or volume, and mechanical properties remain poorly understood^3,14,15^. While measuring the cell mass is challenging, the simultaneous characterization of cell mechanical properties can hardly be tackled by current technologies with sufficient resolution to describe cellular processes^16,17^.

A variety of promising approaches have been introduced to measure cell mass. Optical approaches include confocal microscopy^18^, fluorescence exclusion^19^, or quantitative phase imaging^20^, while mechanical approaches include suspended microchannel resonators^21–24^, micro-electromechanical systems^17,25,26^, or photothermally actuated microcantilevers^27,28^. Yet, only a few of these technologies can measure the total mass of individual adherent cells at culture conditions and in combination with advanced light microscopy, such as required to monitor cell morphology and state. The recently introduced cell balance (“picobalance”) uses a photothermally actuated microcantilever to measure the total mass of adherent single cells under culture conditions^27^. At millisecond (ms) time and picogram (pg) mass resolution, the balance can monitor cell growth over extended time periods from minutes to days, while recording optical images. The picobalance measures the eigenfrequency (or natural resonance frequency) ${f}_{{{{{{\rm{N}}}}}}\_{{{{{\rm{cant}}}}}}}$ of the microcantilever before and after cell attachment ${f}_{{{{{{\rm{N}}}}}}\_{{{{{\rm{cant}}}}}}+{{{{{\rm{cell}}}}}}}$ from which the apparent mass of the cell ${{m}_{{{{{{\rm{cell}}}}}}}}{*}$ is calculated by ${{m}_{{{{{{\rm{cell}}}}}}}}{\ast}=\frac{{k}_{{{{{{\rm{cant}}}}}}}}{{4\pi }^{2}}(\frac{1}{{f}_{{{{{{\rm{N}}}}}}\_{{{{{\rm{cant}}}}}}+{{{{{\rm{cell}}}}}}}^{2}}-\frac{1}{{f}_{{{{{{\rm{N}}}}}}\_{{{{{\rm{cant}}}}}}}^{2}})$ (Eq. 1)^27^, where ${k}_{{{{{{\rm{cant}}}}}}}$ is the spring constant of the cantilever. To account for the position of the cell along the beam of the microcantilever, ${{m}_{{{{{{\rm{cell}}}}}}}}{\ast}$ is multiplied by a correction factor to receive the total cell mass ${m}_{{{{{{\rm{cell}}}}}}}$ (Supplementary Note 1)^27^. Recently introduced designs that reduce the mass of the microcantilever enable to monitor the mass of single adherent cells at much-improved accuracy and higher mass sensitivity^29,30^. However, the equation simplifies the cell as a solid mass, while living cells dynamically change shape, adhesion, spreading, and mechanics (e.g., cortex tension, pressure, and stiffness) during the cell cycle^31–34^. Furthermore, the viscoelastic properties of cells depend on the frequency at which they are mechanically probed^28,35–37^. Yet, how the above equation and thus microcantilever-based cell mass measurements depend on the mechanical properties of the biological cell has not been addressed.

Microcantilevers are widely used to characterize the mechanical properties of cellular systems, particularly in combination with atomic force microscopy (AFM)-based imaging and/or optical imaging^37^. The highly complex and dynamic cellular structures expose heterogeneous mechanical properties. For example, the Young’s modulus of the cytoplasm for different cell lines ranges from 0.1 to 10 kPa^38–45^, while estimations for the actomyosin cortex range from 10 to 1000 kPa^28,31,35,46,47^. This large spread of moduli depends on the cell type, cell state, and experimental settings. For example, cells in the interphase, adherent, or mitotic state show drastically different morphological and mechanical properties^4,5,32^. Additionally, the Young’s modulus depends on the size and shape of the cantilever probe, the cellular location at which the mechanical properties are probed, the force and depth of the indentation applied, as well as the frequency (e.g., velocity) and properties (e.g., spring constant or sensitivity) of the microcantilever^37^. Therefore, depending on the experimental settings, often only a part of the cell is examined (e.g., cell membrane, actomyosin cortex, nucleus, or intermediate filaments), whereas methods that can measure the mechanical properties of the entire cell are rare^48^.

The eigenfrequency is typically considered the most critical mechanical property of a system and describes the frequency at which the system absorbs the most mechanical energy (amplitude) and changes its mechanical response (phase). In complex and viscous cellular systems, however, the amplitude and phase response are determined not only by the eigenfrequency but also by damping effects. This damping or energy loss of the mechanically perturbed cell can be expressed by the quality (Q)-factor. So far, applying such basic mechanical concepts to better describe cellular systems in their entities has only been done to a minor extent, mostly in the context of ultrasound studies^49–51^. Currently, the mechanical response of mammalian cells to externally applied mechanical perturbations is characterized at different frequencies^28,36,52–54^, although hardly any information on the eigenfrequency of cells is available. One theoretical study suggests that externally applied vibrational frequencies approaching the expected eigenfrequency of the cell nucleus induce high-frequency strain regimes^50^. Similarly, finite element method (FEM) simulations describe the cumulative effect of cyclic ultrasound pressure on cell behavior^55^.

Here, we introduce a unique assay to monitor the total mass and mechanical response of mammalian cells using photothermally actuated resonating microcantilevers. We start the development of our assay by searching for the physical origin of why resonating microcantilevers under certain circumstances measure too low cell mass. Our results and theoretical simulations, which examine the response of cells to a variety of externally applied mechanical frequencies, lead to fundamental observations and introduce ways to measure the total mass, eigenfrequency, and Q-factor of living cells.

## Results

### After cell attachment, microcantilevers measure too-low mass

**Fig. 1:**
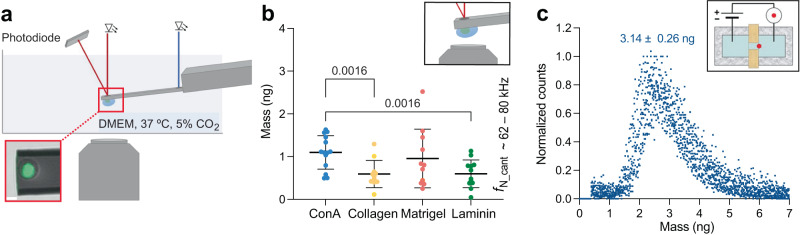
Mass measurements of single HeLa cells adhering to microcantilevers functionalized with different substrates reveal broad mass distributions. **a** Experimental setup to measure the total mass of single cells. The microcantilever is immersed in a dish filled with DMEM culture medium (Methods). The Petri dish is embedded in a controlled environmental system, which enables setting the temperature to 37.0 °C and controlling the pH by using a humidified gas mixture based on synthetic air containing 5% CO_2_. The oscillation frequency of the photo-thermally actuated (blue laser) microcantilever is read out by an infrared laser reflected from the free end of the cantilever onto a position-sensitive photodiode. Simultaneously to microcantilever measurements, optical microscopy images (e.g., DIC, fluorescence) can be recorded (inset; scale bar, 10 µm). **b** Mass measurements of rounded HeLa cells picked up with substrate-coated microcantilevers (*f*_N_cant_ ≈ 62–80 kHz) were conducted ≤1 min after cell attachment to the cantilever. Mean total mass measured on concanavalin A (ConA, *n*_cell_ = 14) 1.10 ± 0.39 ng, collagen (*n*_cell_ = 11) 0.59 ± 0.32 ng, matrigel (*n*_cell_ = 11) 0.96 ± 0.69 ng and laminin (*n*_cell_ = 12) 0.60 ± 0.32 ng. Each dot represents a single cell. Horizontal black lines represent the mean, and error bars the standard deviation. Measurements were taken directly after a cell was attached to the cantilever and the cantilever was raised to measurement position (*t* ≤ 1 min). ${f}_{{{{{{\rm{N}}}}}}\_{{{{{\rm{cant}}}}}}}$ gives the natural eigenfrequency range of the micromachined microcantilevers used. *P* values as obtained from statistical analysis using the two-tailed unpaired *t* test (Welch) are indicated. **c** Average mass of round untreated HeLa cells as estimated from Coulter counter measurements (*n*_repeats_ = 8, each involving >3000 HeLa cells). Measurements using microcantilevers or Coulter device were conducted at approximately the same time point after resuspension, in the same culture medium and temperature (37.0 °C).

To conduct mass measurements, cells must be tightly attached to the photothermally actuated, mechanically oscillating microcantilevers (Fig. 1a)^27^. To investigate this relationship, we decided to characterize the mass of cells adhering to microcantilevers coated with different extracellular substrates. For the attachment of single HeLa cells, the micromachined rectangular microcantilevers were functionalized with concanavalin A (ConA), collagen, matrigel, or laminin (Methods). After confirmation of functionalization (Supplementary Fig. 1), we measured the eigenfrequency ${f}_{{{{{{\rm{N}}}}}}\_{{{{{\rm{cant}}}}}}}$ of each microcantilever in cell culture medium, which ranged from 62 to 80 kHz. Then, the microcantilever was gently approached to a single rounded HeLa cell. Immediately after cell attachment (≤1 min), the cantilever was lifted ≈200 µm above the bottom of the culture plate and the eigenfrequency of the microcantilever with the attached cell ${f}_{{{{{{\rm{N}}}}}}\_{{{{{\rm{cant}}}}}}+{{{{{\rm{cell}}}}}}}$ was measured (Fig. 1b). The eigenfrequency shift $\Delta {f}_{{{{{{\rm{N}}}}}}}={f}_{{{{{{\rm{N}}}}}}\_{{{{{\rm{cant}}}}}}}-{f}_{{{{{{\rm{N}}}}}}\_{{{{{\rm{cant}}}}}}+{{{{{\rm{cell}}}}}}}$ and the position of the cell along the cantilever beam were used to calculate the total mass of the cell ${m}_{{{{{{\rm{cell}}}}}}}\,$ (Eq. 1; Supplementary Note 1)^27^. The total mass of the rounded cells was substrate-dependent, with their mean masses varying by ≈86% from 0.59 ± 0.32 ng to 1.10 ± 0.39 ng (mean ± SD). At this initial stage, cells on collagen- and laminin-coated cantilevers showed the lowest mass and cells on ConA-coated cantilevers the highest mass.

To estimate the cell mass before attachment to the cantilever, we conducted Coulter counter measurements and determined the diameter of suspended round HeLa cells (Fig. 1c). The total mass of round HeLa cells estimated from the calculated cell volume and cell density $\rho$ of 1.06 g cm^–3^ ^56^ was 3.14 ± 0.26 ng (mean ± SD, *n*_repeats_ = 8), which was considerably higher than the cell masses measured with the microcantilever. The comparison of cell masses determined via the microcantilever and the Coulter counter provides a good reference as shortly after attachment to the cantilever (≤1 min) the HeLa cells appeared morphologically very similar to their suspended, rounded state (Fig. 1a). However, the mass measured with the microcantilever differed from the mass estimated through the Coulter counter by a factor of ≈3–5. Because cells cannot be assumed to regulate mass of this magnitude within 1 min, we conclude that the cell mass measured with the microcantilever, regardless of the substrate, is too low and lies outside the expected mass range.

### Adherent cells increase mass over time

**Fig. 2:**
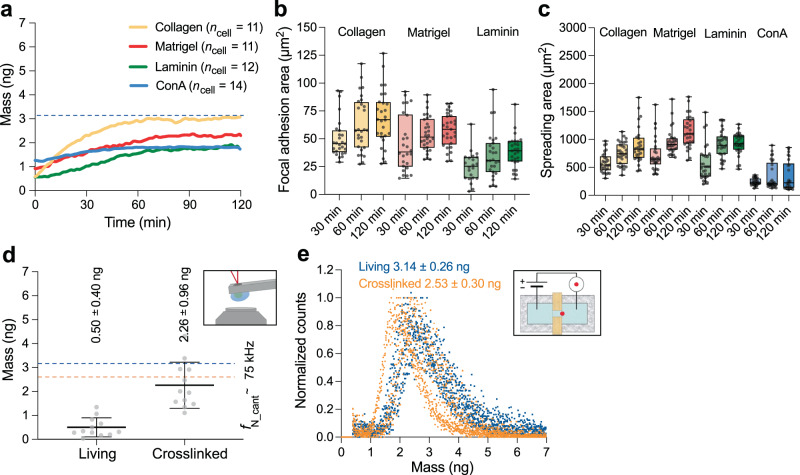
HeLa cells increase mass depending on substrate and reach reference values after chemical fixation. **a** Growth curves of single HeLa cells on four different substrates. Each mass curve represents the mean total mass of *n*_cell_ = 11–14 single cells. Individual mass curves are shown in Supplementary Fig. 3. The mean total cell mass as estimated from Coulter counter measurements is indicated as dashed blue line. **b** Focal adhesion area measured via labeling the focal adhesion-associated protein paxillin indicates the adhesion strengthening of HeLa cells on collagen, matrigel and laminin. Dots represent single cells (*n*_cell_ = 22–30 for each experimental condition). **c** Depending on the substrate, HeLa cells increase their spreading area differently over time. Dots represent single cells (*n*_cell_ = 29–30 for each substrate and time point). Box plots display the interquartile range (box), the median (black line), and whisker (minimum to maximum values). Mean values, standard deviations and statistical comparisons are given in Supplementary Tables 1 and 2 for (**b**) and Supplementary Tables 3 and 4 for (**c**). **d** Chemical crosslinking of cells adhering to ConA-coated microcantilevers increases the measured mass values close to the reference estimated from Coulter counter measurements. Dashed lines indicate the mean total mass of living (blue) and crosslinked (yellow) cells from Coulter counter measurements (**e**). The cell mass was measured within ≤1 min after pick up with the microcantilever. After initial mass measurements (“living”), the cells were chemically crosslinked for 30 min with 2% (v/v) glutaraldehyde, followed by a ≈30 min wash in culture medium without glutaraldehyde, and then measured (“crosslinked”). Dots represent single-cell experiments (*n*_cell_ = 12 for each experimental condition). Values represent the mean (black line) and standard deviation (error bar). **e** Cell mass estimated from Coulter counter measurements showing round living (blue, data taken from Fig. 1c) and chemically crosslinked (2% (v/v) glutaraldehyde, yellow) HeLa cells suspended in culture medium. For both experimental conditions, eight independent experimental repeats (*n*_repeats_ = 8) are shown each involving >3000 HeLa cells.

Microcantilevers have been speculated to require cells to be firmly attached to capture their mass^26,27^. Upon attachment to a substrate, cells strengthen adhesion for ≈60–90 min, which depends on the substrate^57,58^. During this time, cells are expected to regulate substrate-specific growth^59,60^. Consequently, we investigated how the time-dependent adhesion strengthening and growth of cells on different substrates coincide with the cell mass measured by the microcantilever. Thus, we monitored the mass of individual HeLa cells adhering to functionalized microcantilevers for 120 min after attachment (Fig. 2a; Supplementary Figs. 2 and 3). Cells adhering to collagen-, matrigel-, laminin-, or ConA-functionalized cantilevers increased mass differently. However, most cells did not reach the mean mass of suspended cells estimated with the Coulter counter (Fig. 2a). An exception were cells adhering to collagen-functionalized microcantilevers, which increased mass from ≈0.59 ng to ≈3.04 ng within 120 min. Because for all substrates and attachment times ≥30 min HeLa cells adhered firmly to the functionalized cantilevers, the results indicate that the apparent mass discrepancies measured by oscillating microcantilevers originate from other effects than loose adhesion.

### Cell mass and adhesion strengthening correlate imperfectly

To further explore possible correlations between cell adhesion strength and cell mass measured by microcantilevers, we quantified focal adhesion areas by fluorescently staining the focal adhesion-associated protein paxillin (Fig. 2b; Supplementary Fig. 4). Because HeLa cells attaching to ConA do not form focal adhesions, we quantified only the focal adhesion areas of cells adhering to collagen, matrigel, or laminin. Although HeLa cells adhering to collagen and matrigel showed similar focal adhesion areas and the highest masses, on laminin they formed considerably smaller adhesion areas and showed lower masses at all time points from 30 to 120 min after attachment. Consequently, cell mass and focal adhesion area were correlated (Supplementary Fig. 5a). However, at 120 min, except for collagen, the cell masses were considerably below the mean cell mass estimated from Coulter counter measurements (Fig. 2a), suggesting that adhesion is not the only factor for an accurate microcantilever-based mass readout.

Although focal adhesion strengthening cannot be expected on ConA, we measured that cells adhering to ConA increase mass over time (Fig. 2a). Because ConA binds to extracellular sugars on the cell surface, we hypothesize that HeLa cells strengthen adhesion by increasing the contact area with the substrate, which led us to quantify the substrate-dependent spreading areas over time. HeLa cells showed similar spreading areas on collagen, matrigel, and laminin, while the cell spreading area on ConA was smallest and increased minimally (Fig. 2c). While the larger cell spreading areas on collagen and matrigel were correlated with higher cell masses (Fig. 2a), cells on ConA showed higher masses than cells on laminin at ≤60 min and similar masses at 120 min after attachment despite considerably smaller spreading areas for cells on ConA. Consequently, the cell spreading area and mass poorly correlated (Supplementary Fig. 5b).

In summary, we find a correlation between cell focal adhesion areas and cell mass, and a negligible correlation between cell spreading and cell mass. This indicates that cell adhesion plays a role in microcantilever-based cell mass measurements. However, the discrepancy between expected and measured cell mass suggests that this role is limited and that other, possibly more important factors may explain the missing mass in our measurements.

### Correct cell mass after mechanical stiffening

We next reasoned that the attached cell, with its compartments having different viscoelastic properties, might not entirely follow the mechanical oscillations of the microcantilever in phase, such as expected from an attached solid mass (Eq. 1). In such a case, the microcantilever would read out only a fraction of the cell mass, presumably of the cellular compartments moving in phase with the cantilever, while the mass of other parts of the cell is insufficiently detected. We tested this hypothesis by measuring the mass of chemically crosslinked and thus mechanically stiffened HeLa cells (Fig. 2d). In our experiments, we first measured the mass of rounded HeLa cells, which had been freshly attached (*t* ≤ 1 min) to ConA-coated cantilevers. The cells were then chemically crosslinked with 2% (v/v) glutaraldehyde for 30 min. Crosslinking mechanically stiffened the cells by a factor of ≈3 (Supplementary Fig. 6), which was within the range reported earlier^61,62^. While living HeLa cells showed a mass of 0.50 ± 0.40 ng (*n*_cell_ = 12; mean ± SD), chemically crosslinked cells showed a substantially higher total mass of 2.26 ± 0.96 ng (*n*_cell_ = 12). Coulter counter measurements of suspended and rounded HeLa cells before and after crosslinking showed that the cell mass reduced from 3.14 ± 0.26 ng to 2.53 ± 0.30 ng, respectively (Fig. 2e). This mass reduction of ≈20% is based on the shrinkage of the cell volume^63^. Thus, within the experimental accuracies, the cell mass measured with the microcantilever after mechanical stiffening is in good agreement with the cell mass derived from Coulter counter measurements.

### Roles of cell mass, stiffness, and eigenfrequency

**Fig. 3:**
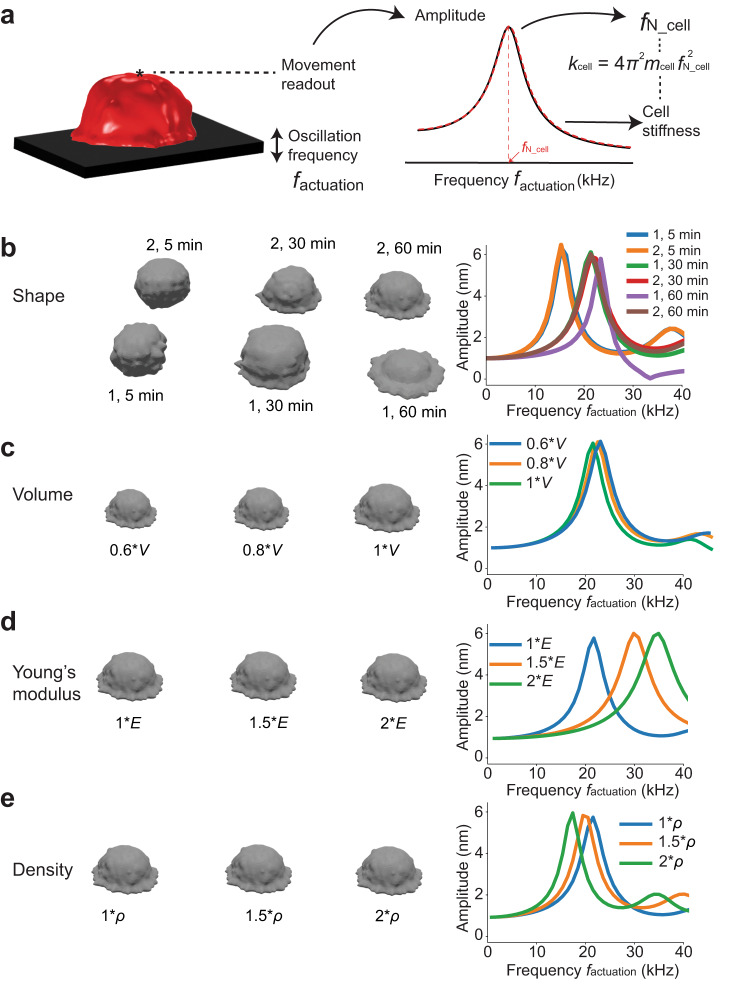
Cell shape, elasticity, and mass affect the stiffness and eigenfrequency of the cell. **a** We simulated an oscillating flat support to which the cell (red) adhered over a frequency range ${f}_{{{{{{\rm{actuation}}}}}}}$ and analyzed the movement at the cell surface opposite to the support (black). The cell geometry was extracted from confocal microscopy images (Supplementary Fig. 7c). Right, by fitting the amplitude response of the cell (red dashed line), the eigenfrequency ${f}_{{{{{{\rm{N}}}}}}\_{{{{{\rm{cell}}}}}}}$ and the stiffness ${k}_{{{{{{\rm{cell}}}}}}}$ of the cell are extracted (Eq. 2). **b** Testing the influence of the cell shape *S* on ${f}_{{{{{{\rm{N}}}}}}\_{{{{{\rm{cell}}}}}}}$ and ${k}_{{{{{{\rm{cell}}}}}}}$. Shown are two examples of cell geometries (1 and 2) determined at adhesion times 5, 30, and 60 min. Cell volumes were normalized to 3500 µm^3^. Right, amplitude response of the cell surface opposite to the support. For ${f}_{{{{{{\rm{N}}}}}}\_{{{{{\rm{cell}}}}}}}$ values see Supplementary Table 5. **c** Testing the influence of the cell volume $V$ on ${k}_{{{{{{\rm{cell}}}}}}}$. We simulated 60%, 80%, and 100% of the corresponding cell volume determined at 60 min in (**a**). Right, amplitude response of the cell surface opposite to the support. **d** Testing the influence of Young’s modulus *E* on ${f}_{{{{{{\rm{N}}}}}}\_{{{{{\rm{cell}}}}}}}$ and ${k}_{{{{{{\rm{cell}}}}}}}.$ We simulated the cell at 60 min (**a**) assuming three different Young’s moduli, namely 100%, 150%, and 200% of the input value *E* = 1.5 kPa. Right, amplitude response of the cell surface opposite to the support. **e** Testing the influence of different densities $\rho$ 1, 1.5, and 2 g cm^–3^ of the cell determined at 60 min in (**a**) on ${f}_{{{{{{\rm{N}}}}}}\_{{{{{\rm{cell}}}}}}}$ and ${k}_{{{{{{\rm{cell}}}}}}}$. Right, amplitude response of the cell surface opposite to the support.

To better understand how the mechanical stiffening of the cell affects the mass readout using microcantilevers, we performed FEM simulations. First, we 3D imaged HeLa cells adhering to ConA and observed their shape changes within 120 min using confocal microscopy (Supplementary Fig. 7). We used the rendered cell shapes to simulate a cell adhering to a flat support, which was oscillated at 1.5 nm amplitude over a range of frequencies ${f}_{{{{{{\rm{actuation}}}}}}}$ (Fig. 3). To calculate the mechanical stiffness (i.e., spring constant) of the entire cell via ${k}_{{{{{{\rm{cell}}}}}}}=4{\pi }^{2}{m}_{{{{{{\rm{cell}}}}}}}{f}_{{{{{{\rm{N}}}}}}\_{{{{{\rm{cell}}}}}}}^{2}$ (Eq. 2), where ${f}_{{{{{{\rm{N}}}}}}\_{{{{{\rm{cell}}}}}}}$ is the eigenfrequency of the cell, we extracted the amplitude and phase of the cell surface, opposite to the oscillating support (Fig. 3a).

In each FEM simulation we varied one of four cell mechanical parameters, shape $S$, volume $V$, Young’s modulus $E$, or density $\rho$, while keeping the other parameters constant (Fig. 3b–e). First, we found that varying the cell shape from round (attachment onset) to the more flattened state increased the eigenfrequency of the cell by a factor of $\sqrt{2}$ and hence ${k}_{{{{{{\rm{cell}}}}}}}$ by factor of 2 (Fig. 3b). Therefore, the smallest cellular eigenfrequencies occurred at an early time point (5 min) after cell attachment (Fig. 3b). Second, changing the cell volume by 40% modulated ${f}_{{{{{{\rm{N}}}}}}\_{{{{{\rm{cell}}}}}}}$ and thus ${k}_{{{{{{\rm{cell}}}}}}}$ by maximally 10%, hence we did not investigate this further (Fig. 3c). Third, increasing the Young’s modulus from 1.5 kPa to 3 kPa increased ${f}_{{{{{{\rm{N}}}}}}\_{{{{{\rm{cell}}}}}}}$ by a factor of $\sqrt{2}$ and hence ${k}_{{{{{{\rm{cell}}}}}}}$ by factor of 2 (Fig. 3d). Finally, doubling the cell density, reduced ${f}_{{{{{{\rm{N}}}}}}\_{{{{{\rm{cell}}}}}}}$ by $\sqrt{2}$ but did not affect ${k}_{{{{{{\rm{cell}}}}}}}$ (Eq. 2, Fig. 3e). To summarize, amongst all characterized mechanical parameters, mainly cell shape $S$ and Young’s modulus $E$ affect the cellular eigenfrequency ${f}_{{{{{{\rm{N}}}}}}\_{{{{{\rm{cell}}}}}}}$ and mechanical stiffness ${k}_{{{{{{\rm{cell}}}}}}}$. Due to the linear effect of $E$ on ${k}_{{{{{{\rm{cell}}}}}}}$, a drastic change in ${k}_{{{{{{\rm{cell}}}}}}}$ based only on changes of $E$ is unlikely, although $E$ might change during cell spreading and cell cycle^64,65^. However, a doubling of ${k}_{{{{{{\rm{cell}}}}}}}$ based on cell shape changes upon transiting from a round to a more flattened state can be expected within one hour (Figs. 2c, 3b; Supplementary Fig. 7)^66^.

We then investigated the role of the cell mass in the accuracy of microcantilever-based mass measurements. Cell size is a good proxy for cell mass. We, therefore, divided the cell mass ${m}_{{{{{{\rm{cell}}}}}}}$ measured using oscillating microcantilevers by the volumetrically expected cell mass ${V}_{{{{{{\rm{optical}}}}}}}*\rho$ and plotted the result against the cell diameter (Supplementary Fig. 8). We found a correlation of –0.93 for the binned data, indicating that heavier cells give less accurate mass measurements. With these results, both cell mass (Supplementary Fig. 8) and stiffness (Fig. 2d, Supplementary Fig. 6) seem to affect the microcantilever-based mass readout. Because the eigenfrequency of the cell connects both parameters ${{k}_{{{{{{\rm{cell}}}}}}}/{m}_{{{{{{\rm{cell}}}}}}} \sim f}_{{{{{{\rm{N}}}}}}\_{{{{{\rm{cell}}}}}}}^{2}$ (Eq. 2), we hypothesize ${f}_{{{{{{\rm{N}}}}}}\_{{{{{\rm{cell}}}}}}}$ to be a major determinant of the accuracy of microcantilever-based cell mass measurements.

### Testing the eigenfrequency hypothesis

**Fig. 4:**
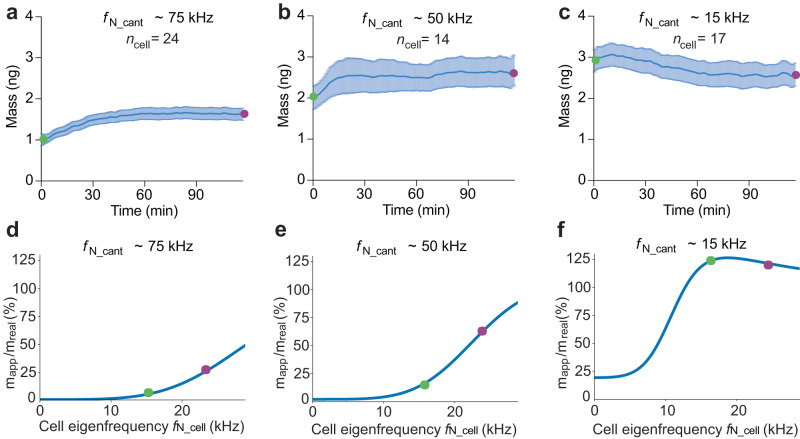
Micromechanical mass readout of single HeLa cells depends on the eigenfrequencies of microcantilever and cell. **a**–**c** 120 min long mass measurements of HeLa cells adhering to ConA-coated microcantilevers, probed at different eigenfrequencies of the cantilever ${f}_{{{{{{\rm{N}}}}}}\_{{{{{\rm{cant}}}}}}}$. **a** ${f}_{{{{{{\rm{N}}}}}}\_{{{{{\rm{cant}}}}}}}$ ≈ 65–95 kHz. **b** ${f}_{{{{{{\rm{N}}}}}}\_{{{{{\rm{cant}}}}}}}$ ≈ 45–50 kHz. **c** ${f}_{{{{{{\rm{N}}}}}}\_{{{{{\rm{cant}}}}}}}$ ≈ 15 kHz. Average curves are presented as mean (blue line) and standard deviation (light blue area) of *n*_cell_ independent cells measured. Green and violet dots represent the start and end states of the measurements, respectively. All experiments were carried out under cell culture conditions. **d**–**f** Lumped-mass model simulations providing the percentage of the real cell mass experimentally measured with microcantilevers having different eigenfrequencies ${f}_{{{{{{\rm{N}}}}}}\_{{{{{\rm{cant}}}}}}}$ in (**a**–**c**) and for increasing cell eigenfrequencies (blue lines, Methods). The increasing eigenfrequency of the cell is based on the dependency of the eigenfrequency ${f}_{{{{{{\rm{N}}}}}}\_{{{{{\rm{cell}}}}}}}$ on the change in cell shape (Fig. 3b). The eigenfrequency of the cell as experimentally determined (see **a**–**c**) at the beginning of the cell mass measurement (*t* ≤ 1 min) is marked with a green dot and the presumed end state with a violet dot (*t* ≈ 120 min). ${m}_{{{{{\rm{app}}}}}}$ represents the cell mass measured by the microcantilever and ${m}_{{{{{\rm{real}}}}}}$ the mean cell mass of 3.14 ng derived from Coulter counter measurements (Fig. 2e).

In principle, our simulations suggest that a cell having an eigenfrequency ${f}_{{{{{{\rm{N}}}}}}\_{{{{{\rm{cell}}}}}}}$ below ${f}_{{{{{{\rm{N}}}}}}\_{{{{{\rm{cant}}}}}}}$ cannot fully follow the mechanical movement of the oscillating microcantilever, and this effect affects the micromechanical cell mass measurements. To test this hypothesis, we performed 120 min long mass measurements of HeLa cells on ConA-coated cantilevers having lower, middle, and higher eigenfrequencies of ${f}_{{{{{{\rm{N}}}}}}\_{{{{{\rm{cant}}}}}}}$ ≈15, 50, and 75 kHz, respectively (Fig. 4a–c). For cantilevers having eigenfrequencies of ≈50 and 75 kHz, we observed a time-dependent increase in cell mass (Fig. 4a,b), which can be explained by an increasing ${f}_{{{{{{\rm{N}}}}}}\_{{{{{\rm{cell}}}}}}}$ such as modeled via lumped mass simulations (Fig. 4d,e). Furthermore, at the beginning of our microcantilever-based mass measurements (*t* ≤ 1 min), the average cell mass measured using ≈50 kHz cantilevers was twice higher and even three times higher for ≈15  kHz cantilevers compared to measurements using ≈75 kHz cantilevers. Interestingly, we observed that the cell mass measured using ≈15 kHz cantilevers decreased slightly over time (Fig. 4c). However, all cell mass trajectories can be explained by the lumped mass model (Fig. 4d–f), when accounting for cell shape-induced changes over time (Fig. 3b). Additionally, increasing the cell stiffness increases the cellular eigenfrequency (Fig. 3d). Thus, the eigenfrequency hypothesis suggests a relationship between the eigenfrequencies of the cell ${f}_{{{{{{\rm{N}}}}}}\_{{{{{\rm{cell}}}}}}}$ and of the cantilever ${f}_{{{{{{\rm{N}}}}}}\_{{{{{\rm{cant}}}}}}}$, which must be considered to properly measure the cell mass with oscillating microcantilevers. Moreover, considering that, under certain conditions, cell mass and stiffness affect the microcantilever-based mass readout, ${f}_{{{{{{\rm{N}}}}}}\_{{{{{\rm{cell}}}}}}}$ can be seen as a representative parameter that describes the mechanical properties of the entire cell.

### Eigenfrequency and correct mass determination of cells

**Fig. 5:**
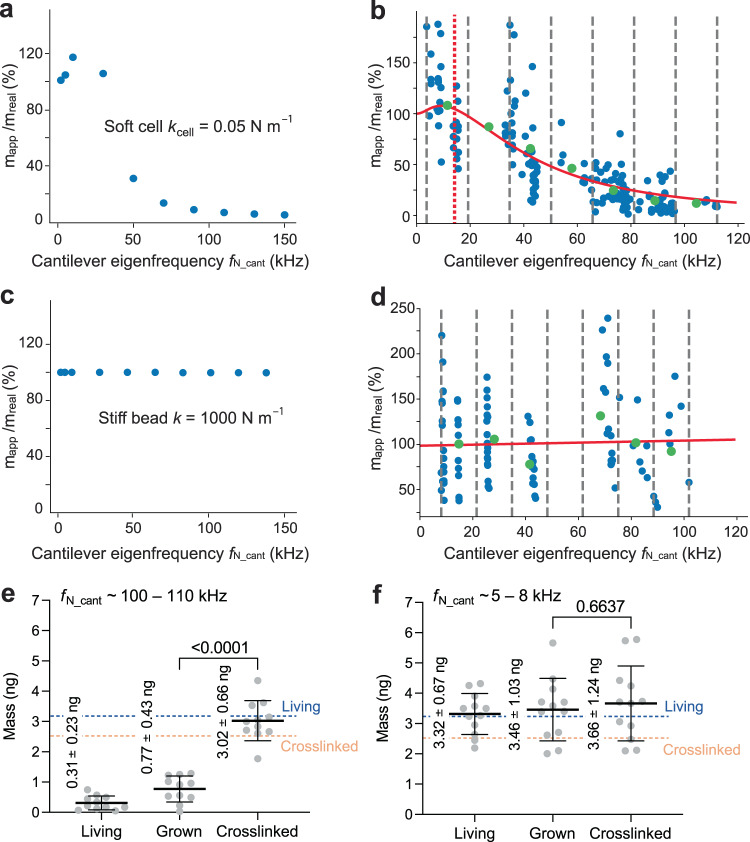
Monitoring the mass, eigenfrequency, and quality (*Q*)-factor of the cell. **a** Relative mass of soft cells revealed by lumped-mass model simulations across microcantilever eigenfrequencies ${f}_{{{{{{\rm{N}}}}}}\_{{{{{\rm{cant}}}}}}}$. **b** Relative mass of living HeLa cells adhering to ConA-coated microcantilevers (*t* ≤ 1 min) measured over ${f}_{{{{{{\rm{N}}}}}}\_{{{{{\rm{cant}}}}}}}$. ${m}_{{{{{{\rm{app}}}}}}}$ represents simulated or measured cell mass, ${m}_{{{{{{\rm{real}}}}}}}$ the mean cell mass of 3.14 ng derived from Coulter counter measurements (Fig. 1c). Blue dots represent single cell simulations (*n*_cell_ = 10) or experiments (*n*_cell_ = 178), green dots means of seven equidistant bins (black dashed lines). Fitting (red line) of experimental data by Eq. 3 extracts ${f}_{{{{{{\rm{N}}}}}}\_{{{{{\rm{cell}}}}}}}$ of 14.4 ± 0.4 kHz (dashed red line) and ${Q}_{{{{{{\rm{cell}}}}}}}$-factor of 0.3 ± 0.1. **c** Relative mass of stiff glass beads revealed by lumped-mass model simulations over ${f}_{{{{{{\rm{N}}}}}}\_{{{{{\rm{cant}}}}}}}$. **d** Relative mass of stiff glass beads measured directly after cantilever attachment (≤1 min), with the mean bead mass ${m}_{{{{{{\rm{real}}}}}}}$ (Supplementary Fig. 9d,e). Blue dots represent single bead simulations (*n*_bead_ = 10) or experiments (*n*_bead_ = 108), green dots means of seven equidistant bins. Experimental data is fitted (red line) by Eq. (3). **e**, **f** Mass measurements of living and crosslinked HeLa cells adhering to cantilevers having eigenfrequencies ${f}_{{{{{{\rm{N}}}}}}\_{{{{{\rm{cant}}}}}}}$ above (**e**) and below (**f**) the eigenfrequency of the cell ${f}_{{{{{{\rm{N}}}}}}\_{{{{{\rm{cell}}}}}}}$. Living cells were picked up with ConA-coated cantilevers and their mass measured within 1 min (“living”) and 30 min (“grown”) after attachment. Afterwards, cells were chemically crosslinked for 30 min in 2% (v/v) glutaraldehyde, washed for 30 min in cell culture medium without glutaraldehyde, and measured (“crosslinked”). **e** Mass measurements of living, grown, and crosslinked HeLa cells by microcantilevers having high ${f}_{{{{{{\rm{N}}}}}}\_{{{{{\rm{cant}}}}}}}$ ≈ 100–110 kHz (*n*_cell_ = 12). **f** Mass measurements of living, grown, and crosslinked HeLa cells by microcantilevers having low ${f}_{{{{{{\rm{N}}}}}}\_{{{{{\rm{cant}}}}}}}$ ≈ 5–8 kHz (*n*_cell_ = 12). Dots represent microcantilever-based single cell measurements. Horizontal black lines represent mean, error bars are the standard deviation. Dashed lines give mean mass of living (blue) and crosslinked (orange) cells approximated through Coulter counter measurements (Fig. 2e). *P* values of statistical analysis using the two-tailed unpaired *t* test (Welch) are indicated in (**e**) and (**f**).

Next, we developed a procedure to extract the cellular eigenfrequency ${f}_{{{{{{\rm{N}}}}}}\_{{{{{\rm{cell}}}}}}}$ and Q-factor ${Q}_{{{{{{\rm{cell}}}}}}}$ from our microcantilever-based approach. We first tested a numerical prediction from the lumped mass model for the dependence of the cell mass readout on the cantilever eigenfrequency (Fig. 5a). The dependency of the mass readout of a soft cell on the cantilever eigenfrequency is clearly revealed. We then measured the mass of HeLa cells directly after attachment (≤1 min) to ConA-functionalized cantilevers having different eigenfrequencies ${f}_{{{{{{\rm{N}}}}}}\_{{{{{\rm{cant}}}}}}}$ ranging from 3 to 110 kHz (Fig. 5b). To avoid cell shape-dependent changes of ${f}_{{{{{{\rm{N}}}}}}\_{{{{{\rm{cell}}}}}}}$, we only considered rounded cells. The experimental data are consistent with the simulation, and the predicted non-linear mass readout as a function of the frequency is prominently displayed in the cell mass measurements. Importantly, such behavior was not observed for stiff beads, where the microcantilevers read out a merely constant mass in simulation and experiment (Fig. 5c, d).

Next, we derived an equation of the cell mass readout accuracy $\frac{{m}_{{{{{{\rm{app}}}}}}}}{{m}_{{{{{{\rm{real}}}}}}}}=1+\frac{{f}_{{{{{{\rm{N}}}}}}\_{{{{{\rm{cant}}}}}}}^{2}*\left({f}_{{{{{{\rm{N}}}}}}\_{{{{{\rm{cell}}}}}}}^{2}-{f}_{{{{{{\rm{N}}}}}}\_{{{{{\rm{cant}}}}}}}^{2}\right)}{\left({f}_{{{{{{\rm{N}}}}}}\_{{{{{\rm{cell}}}}}}}^{2}-{f}_{{{{{{\rm{N}}}}}}\_{{{{{\rm{cant}}}}}}}^{2}\right)^{2}+{f}_{{{{{{\rm{N}}}}}}\_{{{{{\rm{cant}}}}}}}^{2}{f}_{{{{{{\rm{N}}}}}}\_{{{{{\rm{cell}}}}}}}^{2}/{Q}_{{{{{{\rm{cell}}}}}}}^{2}}$ (Eq. 3, Methods), where ${m}_{{{{{{\rm{app}}}}}}}$ represents the apparent cell mass measured by the microcantilever and ${m}_{{{{{{\rm{real}}}}}}}$ the cell mass of 3.14 ng derived from the Coulter counter measurements (Fig. 1c). Fitting the cell mass measured with microcantilevers having different eigenfrequencies with Eq. (3), extracts an average ${f}_{{{{{{\rm{N}}}}}}\_{{{{{\rm{cell}}}}}}}$ of 14.4 ± 0.4 kHz and ${Q}_{{{{{{\rm{cell}}}}}}}$ of 0.3 ± 0.1 (Fig. 5b). The relatively low ${Q}_{{{{{{\rm{cell}}}}}}}$ indicates a high viscous damping within the cell. From ${f}_{{{{{{\rm{N}}}}}}\_{{{{{\rm{cell}}}}}}}$ we deduced an average stiffness of ${k}_{{{{{{\rm{cell}}}}}}}$ = 0.03 N m^–1^ (Eq. 2) and an average Young’s modulus of 1.3 kPa (Methods) for the entire HeLa cell. Both values, stiffness and Young’s modulus, relate very well to those published for mammalian cells^37,67,68^.

The reduced mass readout using high eigenfrequency cantilevers, can be intuitively understood as the cell not being able to follow the cantilever movement. From a mechanical point of view, the viscoelasticity and inertia of the heterogeneously composed cell, causes out-of-phase movements when ${f}_{{{{{{\rm{N}}}}}}\_{{{{{\rm{cant}}}}}}}$ is too high. Consequently, only a fraction of the cell mass is detected whereas measurements at low ${f}_{{{{{{\rm{N}}}}}}\_{{{{{\rm{cant}}}}}}}$ should allow proper mass measurements. We thus measured the mass of living cells and of the same cells, which have been mechanically stiffened through chemical crosslinking, using high eigenfrequency (${f}_{{{{{{\rm{N}}}}}}\_{{{{{\rm{cant}}}}}}} > \, \approx \, 100{{{{{\rm{kHz}}}}}}$) and low eigenfrequency (${f}_{{{{{{\rm{N}}}}}}\_{{{{{\rm{cant}}}}}}}\approx 3-9{{{{{\rm{kHz}}}}}}$) cantilevers (Fig. 5e,f). Living cells adhering to high eigenfrequency cantilevers showed too low masses, which considerably increased after mechanical crosslinking with glutaraldehyde (Fig. 5e). Low eigenfrequency cantilevers, in contrast, measured the same cell mass before and after crosslinking (Fig. 5f). These experiments thus highlight that to measure the correct mass of the cell requires the eigenfrequency of the cantilever to be lower than that of the living cell, so that the entire cell can follow the movement of the cantilever.

## Discussion

Cell mass measurements using resonating microcantilevers can provide inconsistent mass values. Used to extract the total cell mass from the shift of the microcantilever eigenfrequency, Eq. 1 simplifies the cell as a rigidly attached point mass to the cantilever. In stark contrast, living cells are viscoelastic, internally distribute mass in different compartments having heterogeneous mechanical properties, and continuously undergo dynamic processes regulating cell shape, adhesion, spreading, tension, pressure, and stiffness^2–4,7,32,34,37^. Consequently, to accurately measure the total cell mass using mechanically resonating sensors, one must consider the mechanical properties of the entire cell, including all its compartments and components^26^. If some cellular compartments or components cannot follow the mechanical movement of the microcantilever, the cell mass will not be detected properly. This is the case if the eigenfrequency of the oscillating microcantilever ${f}_{{{{{{\rm{N}}}}}}\_{{{{{\rm{cant}}}}}}}$ is higher than the eigenfrequency ${f}_{{{{{{\rm{N}}}}}}\_{{{{{\rm{cell}}}}}}}$ of the living cell. However, microcantilevers read out the correct mass of chemically crosslinked and mechanically stiffened cells.

Our experiments and simulations outline how to overcome this problem by determining the eigenfrequency of the cell ${f}_{{{{{{\rm{N}}}}}}\_{{{{{\rm{cell}}}}}}}$, which describes the mechanical response of the entire cell including all compartments and components. The eigenfrequency of the cell is not a constant parameter and if the cell mechanically stiffens, e.g., via contraction, cell shape change, or chemical crosslinking (Figs. 2d, 5e,f), ${f}_{{{{{{\rm{N}}}}}}\_{{{{{\rm{cell}}}}}}}$ can increase (Fig. 3b). To determine ${f}_{{{{{{\rm{N}}}}}}\_{{{{{\rm{cell}}}}}}}$ of HeLa cells we used microcantilevers with widely varying eigenfrequencies ${f}_{{{{{{\rm{N}}}}}}\_{{{{{\rm{cant}}}}}}}$. If microcantilevers have a ${f}_{{{{{{\rm{N}}}}}}\_{{{{{\rm{cant}}}}}}}$ >> ${f}_{{{{{{\rm{N}}}}}}\_{{{{{\rm{cell}}}}}}}$, they read out too small cell masses. We observe, experimentally and theoretically, an interesting scenario in the intermediate regime where ${f}_{{{{{{\rm{N}}}}}}\_{{{{{\rm{cant}}}}}}}\approx {f}_{{{{{{\rm{N}}}}}}\_{{{{{\rm{cell}}}}}}}$. Here, the oscillation dynamics of the cell can lead to a mass readout that is too high. However, if ${f}_{{{{{{\rm{N}}}}}}\_{{{{{\rm{cant}}}}}}} \ll {f}_{{{{{{\rm{N}}}}}}\_{{{{{\rm{cell}}}}}}}$, Eq. 1 can be applied to determine the correct cell mass from the microcantilever measurements. Finally, our mass readout allows not only extracting the average eigenfrequency of living cells, but also their average ${Q}_{{{{{{\rm{cell}}}}}}}$, which describes the mechanical damping of the cell. Both, eigenfrequency and ${Q}_{{{{{{\rm{cell}}}}}}}$, present new mechanical parameters in cell biology that reflect the dynamic nature of the cell and provide an exciting new direction for the use of microcantilever-based devices as a tailored mechanical toolbox.

Recently, we introduced shorter microcantilevers to reduce their inertial mass and thus to increase the sensitivity (e.g., mass resolution) at which the total cell mass can be measured^29,30^. More advanced designs that cut out rectangular sections of the microcantilever beam allow to considerable reduce the cantilever mass without the need of shortening the microcantilever, to reduce the laser intensity needed for photothermal actuation of the microcantilever and thus possible effects of phototoxicity, and, importantly, to restrict cell migration in order to minimize measurement errors associated with the cell changing its position along the microcantilever^30^. Such recent advancements of cantilever designs together with the experimental and theoretical results presented here highlight that properties such as the mass sensitivity, photoactuation sensitivity, cell position sensitivity, and eigenfrequency of the microcantilever may in the future be carefully adjusted by specifically designing novel microcantilever dimensions and shapes. Therefore, we believe that the future design of microcantilevers and their properties will be fundamental to detect the cell mass more accurately and at the same time to read out specific mechanical properties of the living cell. For example, one may think of sculpting microcantilevers that can oscillate in different mechanical modes to characterize single cells at different ${f}_{{{{{{\rm{N}}}}}}\_{{{{{\rm{cant}}}}}}}$ in a single experiment, instead of using different cells and cantilevers in subsequent experiments. Such approach could extract ${m}_{{{{{{\rm{cell}}}}}}}$, ${f}_{{{{{{\rm{N}}}}}}\_{{{{{\rm{cell}}}}}}}$ and ${Q}_{{{{{{\rm{cell}}}}}}}$ of single cells and not only population averages.

The experiments presented here show that upon chemical fixation and mechanical stiffening of the cell, high eigenfrequency cantilevers can also measure the mass of the entire cell. Because in our experiments the cell is positioned at the free end of the cantilever and the huge difference between cantilever and cell stiffness, this effect cannot be explained by the cell changing the effective cantilever stiffness (Supplementary Note 1, 2). Rather, our systematic study shows that the cell stiffness, and, more generally, the eigenfrequency and *Q*-factor of the cell impact the way the cell moves with the cantilever. Utilizing this insight, we can hypothesize that if actuated at various higher modes, the cantilever may be used to infer mass, stiffness and other mechanical properties of an individual cell, which may be applied in the future to gain complementary insights on the manifold mechanical properties and responses of living cells. Furthermore, the coupling of our microcantilever-based device to optical microscopy further enlarges the scope of the application, allowing the simultaneous monitoring of cell morphology and state. The principles introduced here to extract and combine cell morphology, mechanical parameters, and mass provide a platform for future applications such as the parametrization of cell mechanical responses and phenotypes. Undoubtedly, such insight will advance our understanding of mechanical responses of cellular systems and lead to new biotechnological applications to mechanically target specific cell types in tissues using resonating microcantilevers such as those currently used in ultrasound treatments^69–71^.

## Methods

### Cell line

The HeLa cell line stably expressing H2B-eGFP/actin-mCherry was kindly provided by A.A. Hyman.

### Cell culture

HeLa cells were cultured in a cell culture medium composed of high-glucose Dulbecco’s modified Eagle’s medium (DMEM) supplemented with 1 mM sodium pyruvate, 4 mM GlutaMax, 10% (v/v) fetal calf serum (FCS), 100 units ml^–1^ penicillin and 10 µg ml^–1^ streptomycin (all Gibco Life Technologies). Cells were cultured in T-25 tissue culture flasks (Jet Biofil, Cat.-No.: TCF012025) and kept at 37 °C with 5% CO_2_ in the atmosphere. Cells were used for a maximum of 20 passages. Cells were tested for mycoplasma contamination.

### Picobalance setup

The microcantilever of the picobalance was photo-thermally actuated using a custom 405 nm laser (Schäfter + Kirchhoff GmbH) with the cantilever motions being read out by a custom 852 nm laser (Schäfter + Kirchhoff GmbH)^27^. The photo-thermally actuating laser was driven using the current control mode with the laser diode temperature being kept constant (25 °C) by a laser diode controller (LDC500, Stanford Research Systems). The infrared laser that detected the microcantilever deflection was reflected onto a position-sensitive Si PIN photodiode (S5980 Hamamatsu). To exclude other radiation from reaching the photodiode, we placed a hard-coated bandpass filter (Edmund optics) in front of the photodiode. The picobalance was combined with an inverted optical microscope (Zeiss Observer.Z1; Zeiss code: 036-40587) equipped with an insert Plan-Apochromat 10×/0.3 objective and a digital Hamamatsu camera (Orca Flash 4.0 Cat.-No. C11440 which allowed the simultaneous acquisition of, for instance, differential interference contrast (DIC) or fluorescence images of cells during the mass measurements.

A home-built environmental control system^72^ was used to provide cell culture conditions throughout all mass measurements. This controlled environmental system consists of two concentric chambers. The inner one contains the Petri dish and the outer one is supplied with a humidified gas mixture containing 5% CO_2_, which regulates the atmosphere and pH within the inner chamber. In this way, evaporation of the cell culture medium is minimized and long-term measurements are possible.

For all cell measurements, the sample stage temperature was set to 37 °C. To avoid large temperature gradients, the laboratory temperature was set to 25 °C while the experimental chamber hosting the picobalance was set to 35 °C. This intervention reduced equilibration times required to reduce the thermal drift and fluctuations in laser power, which have to be kept at a minimum to operate the picobalance at stable, drift-free conditions.

### Microcantilever-based cell mass measurements

A Petri dish (Ibidi, Cat.-No: 80136) containing 1.5 ml pre-warmed cell culture medium was placed in the environmental control system of the picobalance. The microcantilever was fully immersed in liquid and both lasers were positioned to optimize the photo-thermal actuation and readout signals. After this, a frequency sweep was performed to determine the eigenfrequency of the microcantilever before cell attachment. Subsequently, the cultured HeLa cells were trypsinized (0.25% trypsin-EDTA, Gibco; Cat.-No. 25200056) and after a recovery time of ≈15 min^73^ in cell culture were added to the Petri dish. For mass measurements, regularly round-shaped and sized (≈16–20 µm diameter) cells were selected. After localizing a rounded cell at the bottom of the Petri dish, the motorized stage of the optical microscope was lowered until the microcantilever gently touched the cell. Upon attachment of the cell to the microcantilever, the cantilever was vertically retracted by ≈200 µm from the bottom of the Petri dish. This vertical retraction was needed to avoid the hydrodynamic coupling of the cantilever and Petri dish^74^. After retraction, the cantilever was oscillated over a range of frequencies (“frequency sweep”), to determine the eigenfrequency of the cantilever with the attached cell, ${f}_{{{{{{\rm{N}}}}}}\_{{{{{\rm{cant}}}}}}+{{{{{\rm{cell}}}}}}}$. From this point onwards, consecutive frequency sweeps of the microcantilever were repeated every ≈10 s. This continuous mode was used for all long-term mass measurements. Additionally, optical microscopy images were recorded every 5 min during the experiment.

### Cell mass measurement analysis

For data analysis, only non-dividing cells showing minimal movements on the microcantilever were considered. For analysis, the cell position on the microcantilever was localized for each time frame and the cell mass was corrected as described^75^ to estimate the total cell mass. All mass experiments were analyzed with an in-house developed software (pyIMD, version 0.1.3)^76^.

The photo-thermal actuation of the microcantilever caused a mean oscillation amplitude of 0.16 nm calculated from *n*_exp_ = 33 experiments using microcantilevers of eigenfrequencies ${f}_{{{{{{{\rm{N}}}}}}\_}_{{{{{{\rm{cant}}}}}}}}$ = 30–90 kHz and converted from millivolts to nanometers using an acquired average optical lever sensitivity of 7.77 ± 2.20 * 10^7 ^V m^–1^ derived from *n* = 20 individual repeats of extracting the slope of the deflection curve when pressing the microcantilevers against the glass bottom of a Petri dish. The amplitudes in millivolts are then divided by the average optical lever sensitivity to get amplitudes in nanometers. For comparison, the mean oscillation amplitude for microcantilevers of ${f}_{{{{{{{\rm{N}}}}}}\_}_{{{{{{\rm{cant}}}}}}}}$ = 30–90 kHz was 1.62 nm (*n*_exp_ = 15), using an average optical lever sensitivity of 3.38 ± 2.03 * 10^7 ^V m^–1^ derived from *n* = 4 individual repeats.

### Coulter counter measurements

The average diameter of HeLa cells in bulk was measured using a Coulter counter (Beckman Coulter Z2, Beckman Coulter Inc.) with an aperture of 100 µm. The cell diameter range was set from 9 to 24 µm. Before and between each measurement, the Coulter counter aperture was flushed with an electrolyte solution (Isoton II diluent, Beckman Coulter). For one measurement, a cuvette containing 10 ml electrolyte solution and 500 µl of suspended HeLa cells (≈5 × 10^5^ cells ml^–1^) in culture medium was placed on the apparatus. The cell diameter distribution obtained by the Coulter counter measurement was used to calculate the cell volume distribution. Thus, the suspended cells were assumed to adopt a spherical shape. The cell volume distribution was then used to calculate the total mass distribution of the cell by assuming a constant density of the cell of 1.06 g cm^−3^ ^56^. For statistical analysis, each bulk cell diameter distribution was plotted as a cell mass distribution. The mean total mass and standard deviation of all distributions were taken to compare the populations of living and crosslinked cells.

### Chemical crosslinking experiments

A Petri dish (Ibidi) containing 1 ml pre-warmed (37 °C) cell culture medium was placed in the picobalance controlled environmental system. The eigenfrequency of the microcantilever was determined, and the cell was picked up as described above. For chemical crosslinking, 500 µl cell culture medium containing 25% (v/v) glutaraldehyde (Sigma) was added to the Petri dish until a concentration of 2% (v/v) glutaraldehyde was reached. After 30 min of incubation, the Petri dish was replaced by a Petri dish containing 1.5 ml of glutaraldehyde-free cell culture medium. A washout period was necessary because the glutaraldehyde changed the viscosity and refraction index of the medium, which in turn affected the mass measurements. Hence, “crosslinked” values (Figs. 2d, 5e,f) refer to the mass measured after 30 min in cell culture buffer in the absence of glutaraldehyde. For Coulter counter measurements, glutaraldehyde was added to cells suspended in cell culture medium until a concentration of 2% (v/v) glutaraldehyde was reached and incubated in an Eppendorf tube for 30 min on a thermomixer (Eppendorf, ThermoMixer F1.5) at 400 rpm, 37 °C before measurement.

### Glass bead experiments

Glass beads (Kisker-Biotech, PSI-15.0) with a nominal diameter of 15 µm were picked up with the microcantilever as done for cell experiments. Given the large size variation (Supplementary Fig. 9a,b), beads with diameters ≈15–25 µm were selected using optical microscopy (DIC) coupled to the picobalance (Fig. 5d). After experiments, the acquired optical images were analyzed by Image J (2.3.0/1.53q), using the circle selection tool to estimate the circumference and diameter of the beads. Assuming the beads were spheres, the diameter was used to calculate their volume, which multiplied by the bead density of 1.8 g cm^–3^ (Kisker-Biotech) provided the bead mass.

### Scanning electron microscopy (SEM) of glass beads

10 µl of glass beads (Kisker-Biotech, PSI-15.0) diluted in PBS were distributed on a carbon sticker mounted on an aluminium sample holder and air dried for 4 h. The beads were sputter coated with a Pt/Au alloy and imaged with a Versa 3D microscope (Thermofisher) at an accelerating voltage of 5 kV. For mass estimations from SEM images, the measured bead diameters were used to calculate the bead volume assuming spheric beads. A bead density of 1.8 g cm^–3^ (Kisker-Biotech) was used to calculate the bead mass.

### Microcantilever production, functionalization, and calibration

Tipless microcantilevers (Mikromasch, HQ:NSC35/tipless/No Al and HQ:CSC38/tipless/No Al) were milled using a focused ion beam (FIB, FEI Helios NanoLab 650). Geometries, eigenfrequencies (in liquid) and spring constants of milled rectangular-shaped microcantilevers are given in Table 1.

**Table 1:** Dimensions of rectangular microcantilevers milled using a focused ion beam

*f*_N_cant_ [kHz]	Length [µm]	Width [µm]	Thickness [µm]	Spring constant (N m^−1^)
90–110	80	35	2	7.8–11.6 (*n*_cant_ = 15)
60–80	120	45	2	8.1–15.6 (*n*_cant_ = 42)
45	120	35	2	2.3–3.2 (*n*_cant_ = 14)
25	200	35	2	2.2–2.5 (*n*_cant_ = 6)
15	200	35	1	0.2–0.8 (*n*_cant_ = 17)
3–8	250	35	1	0.09–0.3 (*n*_cant_ = 14)

The values were approximated from DIC images of microcantilevers. The nominal cantilever thicknesses were provided by the cantilever manufacturer (Methods). Cantilever eigenfrequencies ${f}_{{N\_cant}}$ were determined in liquid. Cantilever spring constants were determined in air using the Sader method^77^. *n*_cant_ gives the number of cantilevers analyzed.

To study the mass of HeLa cells that adhere to different substrates, we coated the microcantilevers with four different proteins or mixtures thereof. Before functionalization, microcantilevers were cleaned with ultraviolet-ozone (UVO) (Jelight Company Inc., Model No. 42-220) for 15 min and then incubated with the substrate in phosphate-buffered saline (PBS, Gibco) at 4 °C overnight. The substrates and concentrations used for the functionalization of the cantilevers were 0.03 mg ml^–1^ collagen I (PureCol), 2 mg ml^–1^ concanavalin A (Sigma), 50 µg ml^–1^ laminin mix (Sigma) and 2% (v/v) matrigel (Corning). After overnight incubation, the cantilevers were washed and kept in PBS (1X, Gibco) and used immediately or stored at 4 °C for up to 24 h. Assessment of cantilever functionalization was done by adding 1 µl of Alexa 488 N-hydroxysuccinimide (NHS) ester (Thermo Fisher Scientific; Cat.-No.: A20000) to the protein incubation (Supplementary Fig. 1a–d). NHS ester is amine-reactive and conjugates the fluorescent dye to the protein. To ensure that the dye itself does not label the cantilever (negative control), we added 1 µl dye diluted in PBS at 4 °C overnight and showed that there was no labeling without the addition of a substrate protein (Supplementary Fig. 1e). Labeling of the microcantilever substrate by the Alexa 488 dye was evaluated using an inverted microscope (Observer Z, Zeiss) equipped with an LSM 700 (Zeiss) confocal head.

For all experiments, microcantilevers were cleaned after use for 2 × 4 min at room temperature (RT) in 95% sulfuric acid (Sigma; Cat.-No.: 038751.K4) and rinsed in ultrapure water between each step and at the end. Before UVO cleaning, microcantilevers were dried with Kimtech wipes and stored in covered plastic dishes.

The cantilever spring constants were determined in air using the Sader method^77^.

### Immunofluorescence microscopy and quantification

In a 12-well plate with coverslips (VWR, Cat.-No. 631-0153), 3 coverslips per substrate condition were coated with respective proteins at 4 °C overnight. The next day, HeLa Kyoto wildtype cells were seeded on each substrate and crosslinked after 30, 60, or 120 min with 4% (v/v) paraformaldehyde (Thermo Fisher Scientific, Cat. No.: 043368.9 L) for 15 min at RT. Subsequently, cells were permeabilized with 0.2% (v/v) Triton X-100 (Thermo Fisher Scientific)) in PBS at RT and washed with PBS. After blocking in a solution (later referred to as PBT) of PBS supplemented with 3% (w/v) bovine serum albumin (BSA) and 0.1% (v/v) Tween-20 (Sigma, Cat.-No. P1379) for 30 min at RT, coverslips were incubated with 100 µl primary antibody (paxillin, rabbit monoclonal Y113, 1:400; Abcam ab32084) diluted in PBT solution at 4 °C overnight in a humidity chamber. Resuming the next day, the coverslips were rinsed five times for 5 min with PBS and then incubated for 1 h at RT in a humid chamber with 100 µl of secondary antibody (goat anti-rabbit, IgG H & L Alexa Fluor 488, 1:400; Abcam ab150077) diluted in PBT. After being rinsing in PBS again, coverslips were removed from the 12-well plate, excess liquid was drained and coverslips mounted on a clean slide with a small drop of mounting medium (prolong gold antifade mountant, Thermo Fisher Scientific, Cat No: P36930). After mounting, the slides were placed in a dark room for at least 24 h to dry. Before imaging, any excess mounting medium was removed with a tissue soaked in 30% (v/v) ethanol (≥99.8% purity; Honeywell Chemicals) and the coverslips were sealed with nail polish.

An image of a single plane containing the paxillin signal was used for both focal adhesion and spreading area quantification. Representative images for spreading area and focal adhesion area quantification are shown (Supplementary Fig. 4). The in-house created macro to determine respective areas was used with ImageJ (version 2.3.0/1.53q). For the segmentation of cell spreading area and focal adhesions from paxillin signal, RenyiEntropy^78^ and MinError^79^ methods were, respectively, used. During particle analysis, a focal adhesion size range of 0.3–50 µm^2^ was considered. Fluorescence images were taken with a point-scanning confocal microscope (Zeiss LSM980) equipped with a LD C-apochromat 40×/1.1 water immersion objective and a quasar GaAsP-PMT spectral detector. Therefore, a 488 nm actuating diode laser was used at 1% power and a detection range of 491–579 nm was collected by the quasar spectral detector (gain 625 V). The sampling settings were set to 108 nm pixel size; 1.61 µs pixel dwell time; unidirectional scanning; 38 µm pinhole (1 airy unit).

### Microscopy to estimate the cell shape

HeLa Kyoto wildtype cells cultured in an eight-well micro-slide (Ibidi, Cat.-No.: 80826) were chemically crosslinked, extracted and blocked as described above. After rinsing with PBS, the samples were incubated with a 1:1000 dilution of SiR-actin (Spirochrome SC001) in PBT solution for 1 h at RT and mounted as described above. Fluorescence images were taken with a point-scanning confocal microscope (Zeiss LSM980) equipped with an LD C-apochromat 40×/1.1 water immersion objective and a quasar GaAdP-PMT spectral detector. Thereby, a 639 nm actuating diode laser was used at 0.5% power and a detection range of 641–694 nm was collected by the quasar spectral detector (gain 650 V). Sampling settings were set to 89 nm pixel size; 0.66 µs pixel dwell time; unidirectional scanning; line averaging of 2; 23 µm pinhole (0.48 airy units). The Z-stacks of individual cells were taken with an 0.32 µm interval.

Cell segmentation was performed using the surface detection tool in Imaris (version 9.8.2, Oxford Instruments). Since the fluorescent signal is mostly found in the cell membrane, a post-processing step by binary morphology was added to fill the surface object for accurate volume measurements (using the Imaris statistics module). The smoothing parameter of the surface detector was chosen to modulate the details of the cell membrane for downstream analysis. The smoothing was set to be 4x the lateral pixel size of the acquired confocal data. Since Imaris creates very high-count triangle meshes, the final surface object is exported as a VRML2 mesh and imported in Blender (Blender Foundation) where it is down-sampled by 90% using the decimate tool. The simplified mesh (Fig. 3a) was used for FEM simulations.

### FEM simulations

To investigate the effect of cell shape, size, stiffness and density on the cell eigenfrequency FEM simulations were employed using the structural mechanics module of COMSOL (COMSOL Multiphysics GmbH, version 5.5). To this end, the simplified geometries (see Methods: “Microscopy to estimate the cell shape”) were imported. On the flat part of each individual cell that attaches to the glass bottom of the Petri dish or microcantilever, a displacement of 1.5 nm was prescribed at varying frequencies using the frequency sweep study. The cell was modeled as a linear elastic material with isotropic losses ($\eta=0.8$) and with varying Young’s moduli (1.5, 2.3 and 3 kPa)^67,68^, densities (1, 1.5, and 2 g cm^–3^) or volumes (3500, 2800, and 2100 μm^3^). To measure the amplitude and phase of the cell as a response to the displacement employed, a point probe was attached to the top of the cell.

### Lumped mass model simulations of cell mechanical properties

To capture how the mass readout of a single cell is affected by changing the eigenfrequency of the cell ${f}_{{{{{{\rm{N}}}}}}\_{{{{{\rm{cell}}}}}}}$ (Fig. 4), a cantilever lumped mass model was studied, implemented in Python 3.7. The cantilever was modeled as a Kelvin-Voight element using spring constant, mass, and damping coefficient, such that the Q-factor and eigenfrequency of the cantilever matched the experimental values for the different cantilevers. Similarly, the cell was modeled as a point mass which was, however, not rigidly attached to the cantilever, but attached via a spring and damper element, and forming a 2-degree-of-freedom system^26^ with spring constant ${k}_{{{{{{\rm{cell}}}}}}}$ and damping coefficient ${c}_{{{{{{\rm{cell}}}}}}}$. For simulations, the mechanical stiffness of the cell ${k}_{{{{{{\rm{cell}}}}}}}$ was linearly increased from 0 to 0.4 N m^–1^ for each cantilever frequency. ${k}_{{{{{{\rm{cell}}}}}}}$ is composed of the elastic modulus $E$ and the cell shape dependent factor $F(S)$, i.e., ${k}_{{{{{{\rm{cell}}}}}}}=E*F(S)$. The increase of ${k}_{{{{{{\rm{cell}}}}}}}$ for the simulation is motivated by the experimentally observed cell shape change (Fig. 3b; Supplementary Fig. 7). However, the change in shape affects the mechanical stiffness and the damping of the cell ${c}_{{{{{{\rm{cell}}}}}}}=\mu*F(S)$. To simulate the dependency on the cell shape, we therefore assumed a minimal damping of ${c}_{{{{{{\rm{cell}}}}}}}=$2.5 mPa·s, which linearly increased with ${k}_{{{{{{\rm{cell}}}}}}}$. The values are consistent with other values for the extensional viscosity of cells^26^. The presumed end state as observed in the experiments is marked with a violet dot. It corresponds to a shape change based doubling of the mechanical stiffness (Fig. 3). For the extraction of the mechanical properties of the cell ensembles (Fig. 5), we solved the 2-degree-of-freedom system and extracted the apparent shift of the cantilever eigenfrequency $\Delta {f}_{{{{{{\rm{N}}}}}}\_{{{{{\rm{cant}}}}}}}$ as observed when assuming a simple harmonic oscillator. With $\Delta {f}_{{{{{{\rm{N}}}}}}\_{{{{{\rm{cant}}}}}}}$ we yielded: $\frac{{m}_{{{{{{\rm{app}}}}}}}}{{m}_{{{{{{\rm{real}}}}}}}}=1+\frac{{f}_{{{{{{\rm{N}}}}}}\_{{{{{\rm{cant}}}}}}}^{2}*\left({f}_{{{{{{\rm{N}}}}}}\_{{{{{\rm{cell}}}}}}}^{2}-{f}_{{{{{{\rm{N}}}}}}\_{{{{{\rm{cant}}}}}}}^{2}\right)}{\left({f}_{{{{{{\rm{N}}}}}}\_{{{{{\rm{cell}}}}}}}^{2}-{f}_{{{{{{\rm{N}}}}}}\_{{{{{\rm{cant}}}}}}}^{2}\right)^{2}+{f}_{{{{{{\rm{N}}}}}}\_{{{{{\rm{cant}}}}}}}^{2}{f}_{{{{{{\rm{N}}}}}}\_{{{{{\rm{cell}}}}}}}^{2}/{Q}_{{{{{{\rm{cell}}}}}}}^{2}}$ (Eq. 3). This formula was fitted to the data shown in Fig. 5b, providing ${f}_{{{{{{\rm{N}}}}}}\_{{{{{\rm{cell}}}}}}}$ = 14.4 ± 0.4 kHz and ${Q}_{{{{{{\rm{cell}}}}}}}$ = 0.3 ± 0.1. As ${f}_{{{{{{\rm{N}}}}}}\_{{{{{\rm{cell}}}}}}}$ represents an average eigenfrequency, we achieved an average mechanical stiffness ${k}_{{{{{{\rm{cell}}}}}}}$ = 0.03 N m^–1^ using Eq. 2 and the average cell mass of 3.14 ng, which equates $E=1.3{{{{{\rm{kPa}}}}}}$ for the cell shape 5_1 (cell1 at 5 min) (using Comsol).

### Live cell and chemically crosslinked stiffness measurements

To quantify the stiffness change between a live cell and a chemically crosslinked cell, we mounted an AFM (Nanowizard II, JPK Instruments) equipped with a CellHesion module (JPK Instruments) on an inverted microscope (Zeiss ObserverZ). Measurements were performed at 37 °C in complete cell culture medium supplemented with 16.9 mM HEPES (Sigma). V-shaped, tipless cantilevers (NPO-D, Bruker, nominal stiffness *k* = 0.06 N m^–1^) were plasma treated for ≈ 5 min and incubated in ConA (2 mg ml^–1^) in PBS at 4 °C overnight. The spring constant of each cantilever was measured using the thermal-noise method^80^. After this, HeLa cells were removed with trypsin-EDTA 0.25%, spun at 400 *g* for 90 s. Cell pellets were resuspended in 500 µl cell culture medium and allowed to recover for ≈15 min. A small aliquot of suspended cells was then added to the Petri dish (Fluorodish FD35, WPI) in cell culture medium.

Using the AFM, the functionalized cantilever was lowered to attach an individual cell. After ≈2 min to allow the cell to form firm contact with the cantilever and stabilize the AFM drift, the cell was pressed onto the Petri dish with an approach speed of 5 µm s^–1^ until a force of 5 nN was reached and immediately retracted from the substrate at 5 µm s^–1^. The experiment was repeated 15 times per cell with a 20 s waiting time between approach-retract cycles to allow the cell to recover. After probing the mechanical properties of the living cell, glutaraldehyde (final concentration 2% v/v) was added to the culture medium and crosslinking was allowed for 20 min. Subsequently, the glutaraldehyde containing Petri dish was replaced by a second Petri dish containing fresh cell culture medium into which cantilever and crosslinked cell were immersed. After a waiting time of ≈2 min to stabilize for the thermal drift, 15 force–distance curves of the crosslinked cell were recorded using the same settings as for the living cell. The analysis of the recorded force-distance curves was done using the AFM software (JPK data processing software, version spm-4.3.55) and the cell stiffness was extracted as described^37^ by fitting the slope of the approach force-distance curve between 1 nN and 4 nN.

### Statistical analysis

To evaluate statistical significance, data sets were first tested for normal distribution (Anderson-Darling, D’Agostino & Pearson, Shapiro-Wilk, Kolmogorow-Smirnov tests). If the data sets were normally distributed, a parametric unpaired two-tailed *t* test with Welch’s correction was applied. If datasets were not normally distributed, a nonparametric Mann-Whitney *t* test was applied. *ns*, nonsignificant (*P* > 0.05); *P ≤ 0.05; **P ≤ 0.01; ***P ≤ 0.001; ****P ≤ 0.0001. All statistical analyses were performed with Prism.

### Reporting summary

Further information on research design is available in the Nature Portfolio Reporting Summary linked to this article.

### Supplementary information

Supplementary Information

Reporting Summary

### Source data

Source Data

Peer Review File

## Data Availability

The data generated in this study are available within the article and its Supplementary Information files or from the corresponding author upon request. Source data are provided with this paper. The data generated in this study have been deposited in the ETH research collection and is available under 10.3929/ethz-b-000654725.
