# Supplementary material for: Monitoring the mass, eigenfrequency, and quality factor of mammalian cells
Source: Nat Commun. 2024 Feb 26;15:1751. doi: 10.1038/s41467-024-46056-7 (PMC10897412; doi:10.1038/s41467-024-46056-7)
Supplement: Supplementary file 1 — Supplementary Information [file 41467_2024_46056_MOESM1_ESM.pdf]

# Monitoring the mass, eigenfrequency, and quality factor of mammalian cells

## SUPPLEMENTARY INFORMATION

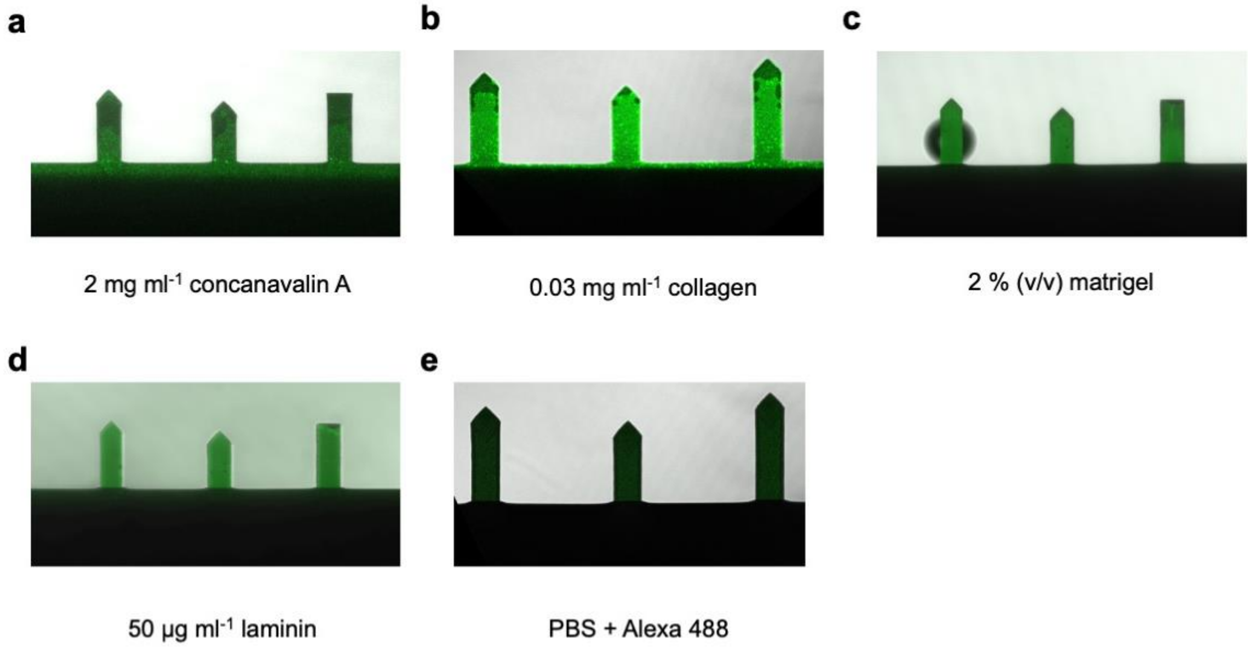


**Supplementary Figure 1. Microcantilever functionalization with different substrates**. Microcantilevers functionalized with **a** concanavalin A (ConA**)**, **b** collagen I, **c** matrigel, **d** laminin and **e** negative control (PBS and Alexa 488 NHS ester). For functionalization the cantilevers were incubated overnight with the desired protein substrate and a fluorescent dye (Alexa 488 NHS ester). Shown are superimposed differential interefence contrast (DIC) and confocal microscopy images of microcantilevers immersed in PBS. The fluorescence signal of the dye added to the respective substrate is shown in green. The experiments were repeated three independent times.

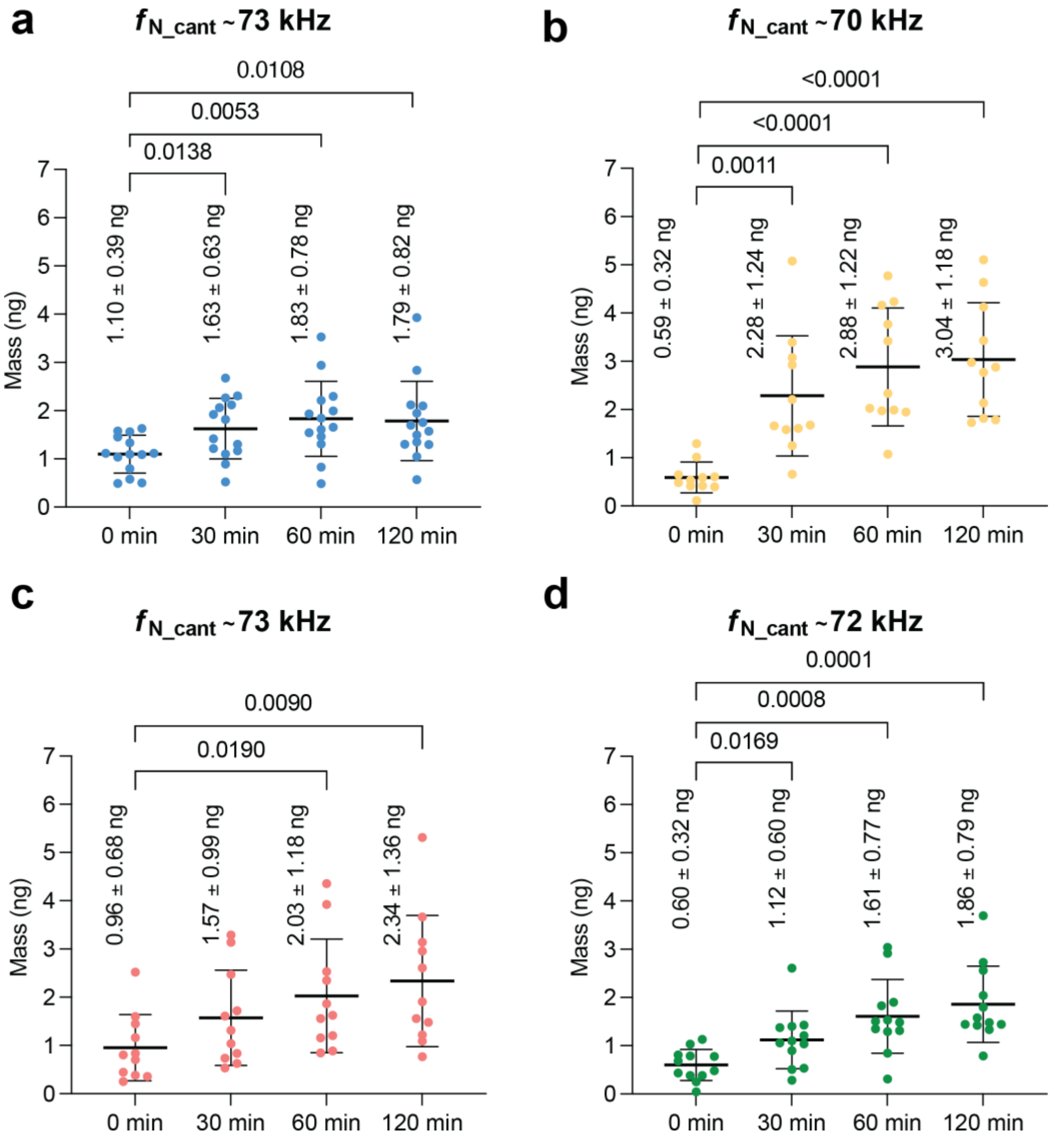


**Supplementary Figure 2. HeLa cells adhering on microcantilevers increase mass differently depending on the substrate functionalizing the cantilever.** The plots show the mass of single HeLa cells adhering to microcantilevers ($f_{N\_cant} \approx 62 - 80$ kHz; dimensions (l x w x t) ≈ 120 x 45 x 2 µm) functionalized with **a** concanavalin A ($n_{cell} = 14$), **b** collagen ($n_{cell} = 11$), **c** matrigel ($n_{cell} = 11$) and **d** laminin ($n_{cell} = 12$) for different time points. All experiments were carried out in cell culture medium under cell culture conditions (Methods). Each dot represents one single cell experiment. Values represent the mean (horizontal black line) and standard deviation (error bars). Statistical analysis using the two-tailed unpaired t-test (Welch) and *P* values are indicated.

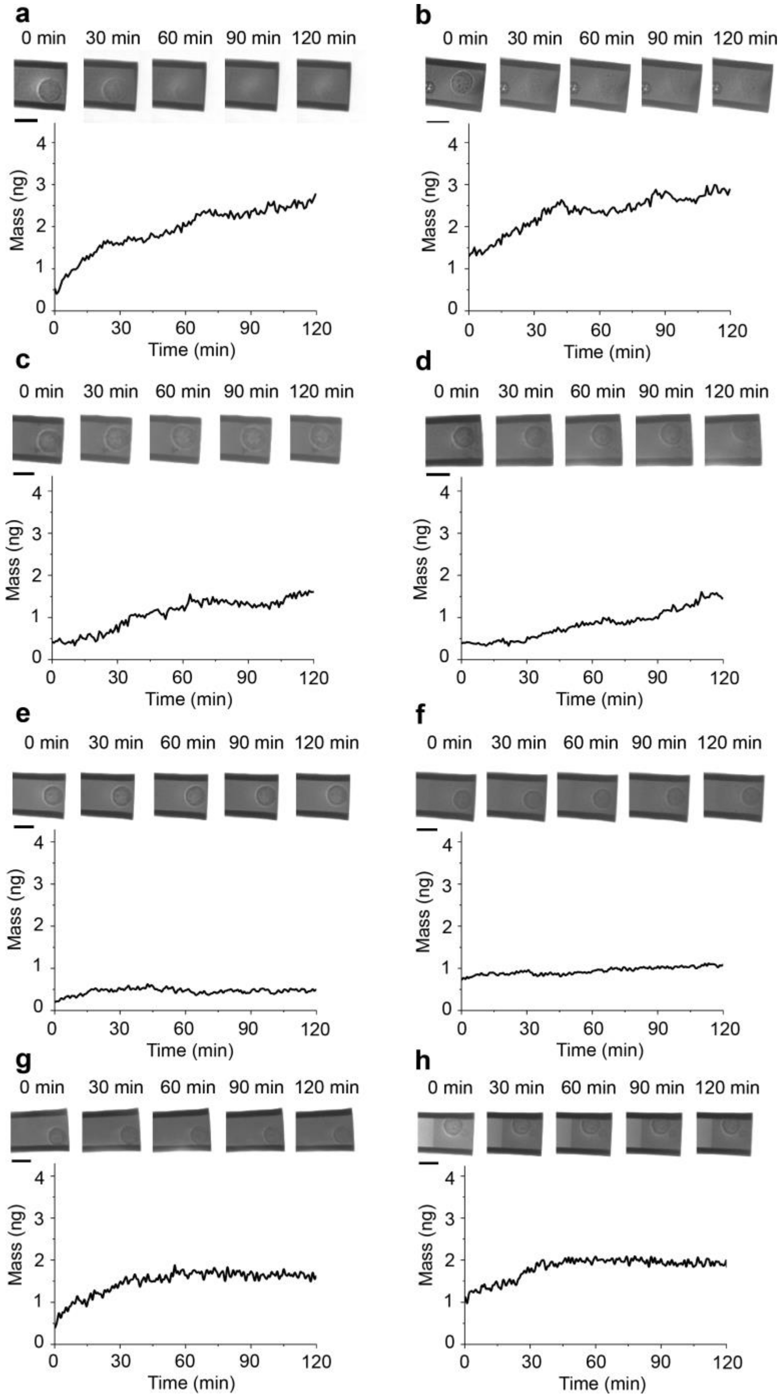


**Supplementary Figure 3. Mass measurements of single cells adhering to rectangular microcantilevers functionalized with different substrates**. Representative mass measurements of HeLa cells on **a, b** collagen I, **c, d** Matrigel, **e, f** laminin, or **g, h** concanavalin A. The cell mass was recorded over 120 min after attaching the cell to a rectangular microcantilever ($f_{N\_cant} \approx 70 - 92$ kHz; 120 x 45 x 2 (l x w x t) µm) in cell culture medium under cell culture conditions. DIC images of the HeLa cell adhering to the microcantilever are shown for each measurement at five different time points. The mass data was analyzed and corrected to account for the cell position and movement on the cantilever using the pyIMD software (Methods). Scale bars, 20 µm.

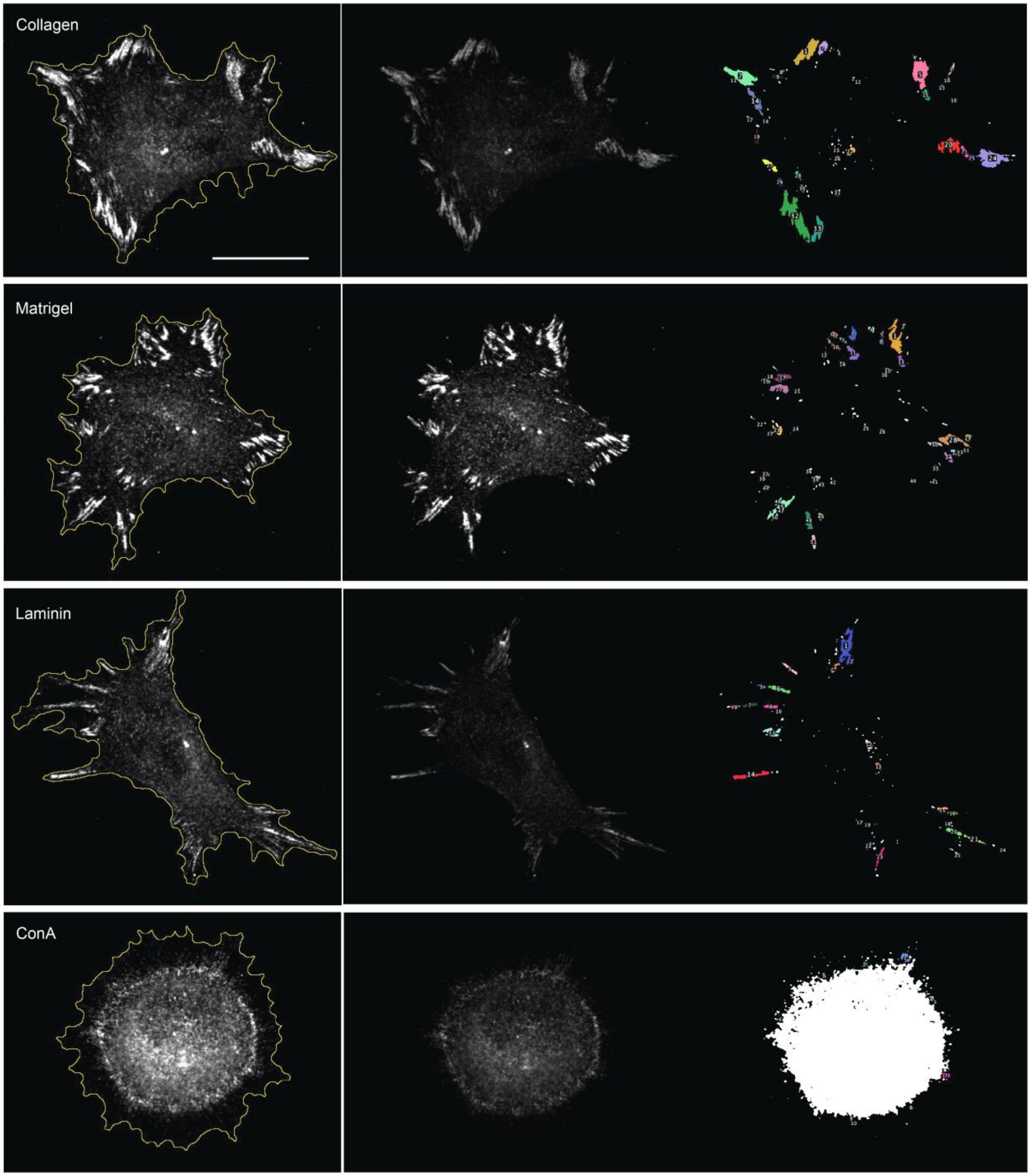


**Supplementary Figure 4. Representative fluorescence microscopy images of paxillin-stained HeLa cells to quantify their spreading (left) and focal adhesion (right) areas on different substrates.** An in-house built macro was used in ImageJ2 (Version 2.3.0/1.53q) to determine the spreading area and the focal adhesion area (Methods). Scale bars, 20 μm.

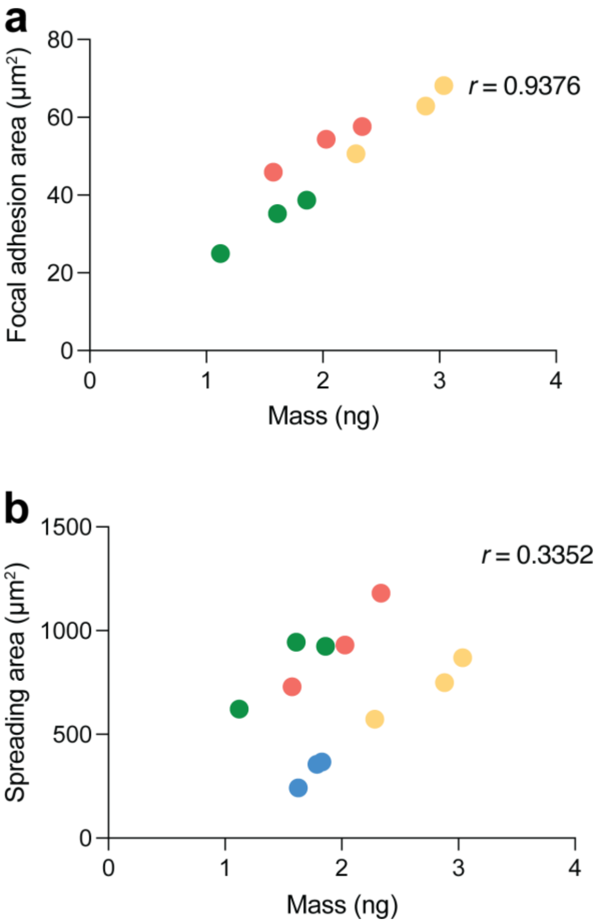


**Supplementary Figure 5. Correlation between focal adhesion area and mean total mass and between spreading area and mean total mass. a**, The mean total mass of HeLa cells adhering to collagen ($n_{\text{cell}}$ = 11, yellow dots), matrigel ($n_{\text{cell}}$ = 11, red dots) and laminin ($n_{\text{cell}}$ = 12, green dots) -functionalized cantilevers after 30, 60 and 120 min of attachment was correlated with mean focal adhesion area determined from HeLa cells ($n_{\text{cell}}$ = 22 – 30 for each substrate) seeded on substrate-functionalized Petri dishes after 30, 60 and 120 min. The correlation between the mean total mass and mean focal adhesion area shows a Pearson coefficient of $r$ = 0.9376 ($P$-value = 0.0002), suggesting a rather strong correlation. **b**, The mean total mass of HeLa cells adhering to collagen ($n_{\text{cell}}$ = 11, yellow dots), matrigel ($n_{\text{cell}}$ = 11, red dots), laminin ($n_{\text{cell}}$ = 12, green dots) and ConA ($n_{\text{cell}}$ = 14, blue dots) -functionalized cantilevers after 30, 60 and 120 min of attachment were correlated with the mean spreading area of cells ($n_{\text{cell}}$ = 29 – 30 for each substrate) seeded on substrate-functionalized Petri dishes after 30, 60 and 120 min. The correlation between the mean total mass and mean spreading area shows a Pearson coefficient of $r$ = 0.3352 ($P$-value = 0.2868), suggesting a very weak correlation. Statistical analysis for determining $P$-values used a two-tailed unpaired t-test (Welch).

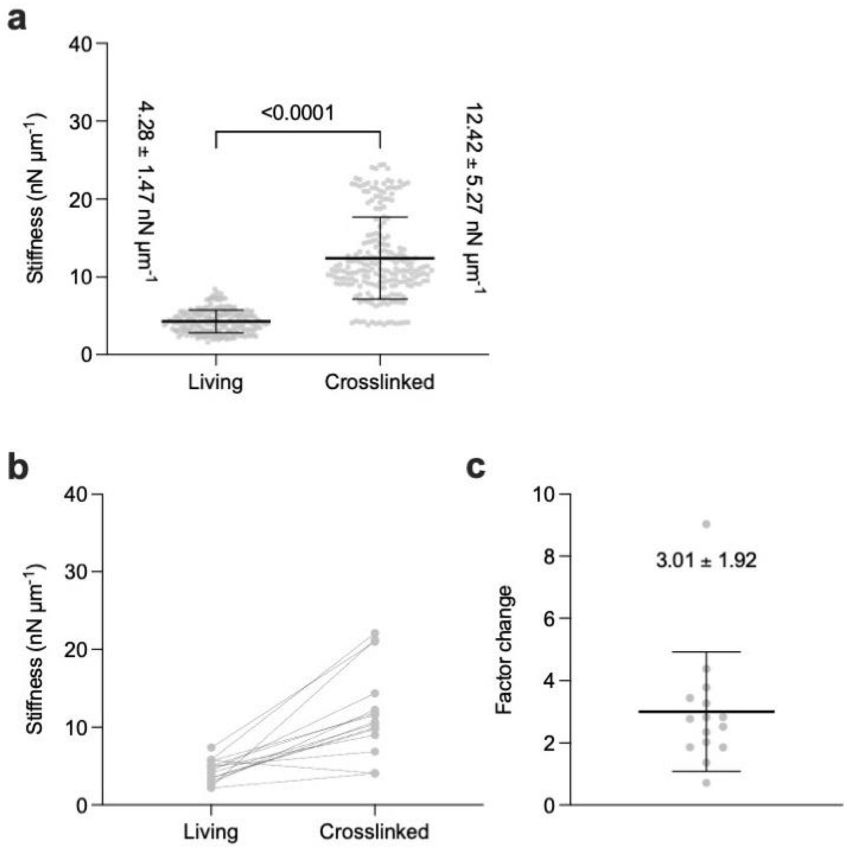


**Supplementary Figure 6. Stiffness of living and chemically crosslinked HeLa cells. a**, Stiffness of living and chemically crosslinked HeLa cells as probed by AFM cantilevers. 14 cells were probed per condition and 15 force-distance curves were taken per cell. The experiments were performed with soft ($k_{cant} \approx 0.04 - 0.14$ N m$^{-1}$) tipless cantilevers. Individual cells were picked up with ConA-functionalized cantilevers and after an attachment period of ≈ 2 min pressed against Petri dishes with a force of 5 nN. After this first mechanical probing of the cellular stiffness, the cells on the cantilever were chemically crosslinked with 2 % (v/v) glutaraldehyde for 20 min under cell culture conditions. Then, the glutaraldehyde-containing cell culture medium was completely exchanged with fresh medium, and the crosslinked cells were mechanically probed again. All experiments were carried out in cell culture medium under cell culture conditions (Methods). Each dot represents the stiffness of one HeLa cell as derived from the force-distance curves. t-test (Mann-Whitney): two-tailed $P$-value < 0.0001. **b**, Average stiffness of HeLa cells before (living) and after crosslinking. Grey dots in represent single cells with the thin dashed lines connecting the living cell to the same cell after crosslinking ($n_{cell} = 14$). **c**, Stiffness change of individual cells (dots) due to chemical crosslinking. Values represent the mean (horizontal black line) and standard deviation (error bars). Force-distance curves were analyzed using the AFM software (JPK data processing software, version spm-4.3.55) and the slope between 1 nN and 4 nN was used to measure the cell stiffness (i.e., force per distance).

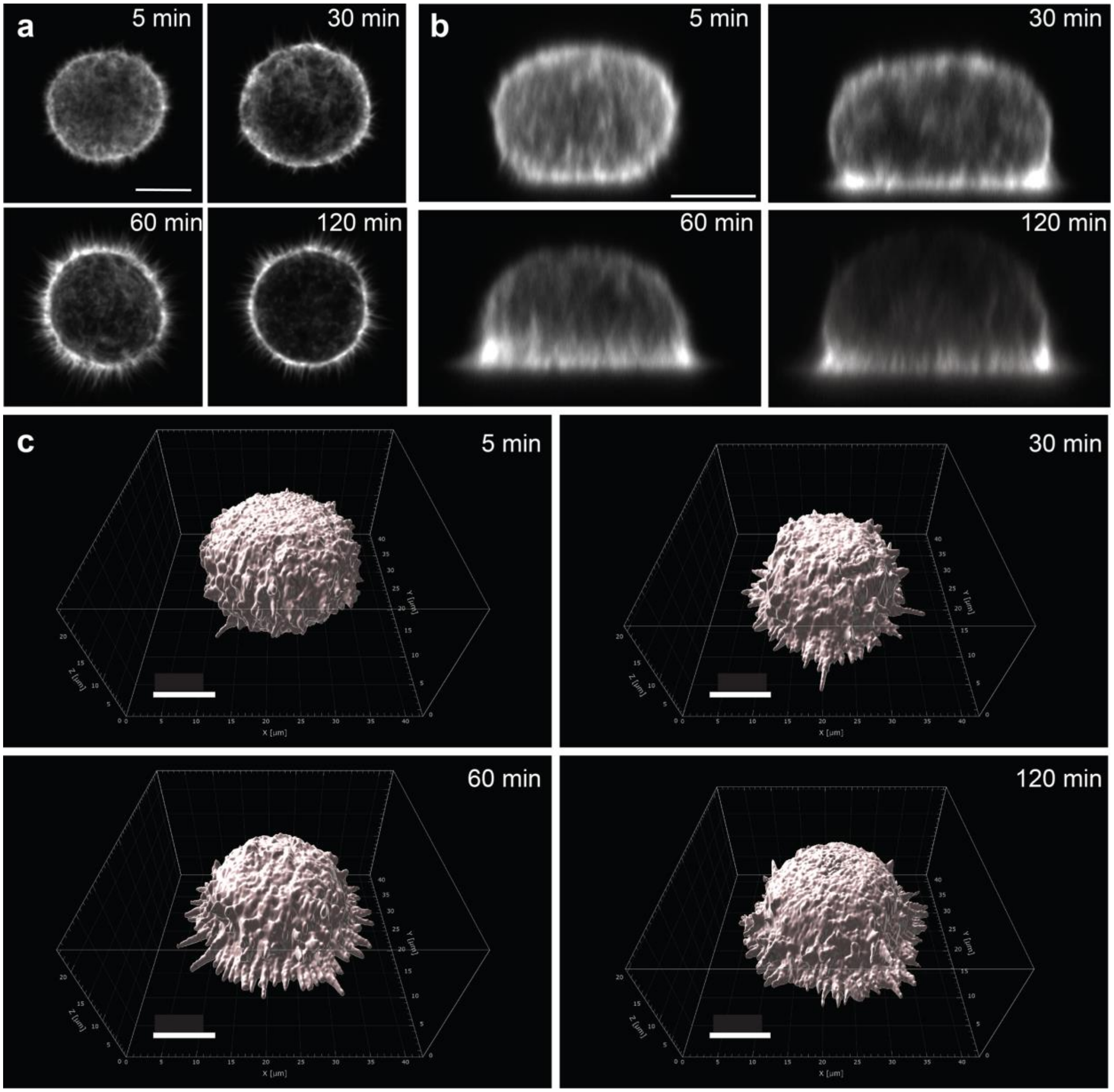


**Supplementary Figure 7. Time-dependent shape changes of HeLa cells adhering to ConA-coated micro-slides (Ibidi).** The cells were chemically crosslinked with 2 % (v/v) glutaraldehyde at four time points from 5 to 120 min and stained with SiR-actin. Imaging was done using confocal microscopy (Methods). **a**, Summed projections of the z-stacks of HeLa cells. **b**, Summed projections of the orthogonal view. Z-stacks were projected using Image J2 (version 2.3.0/1.53q). **c**, 3D reconstructed, rendered, and pre-processed images of HeLa cells used for finite element method (FEM) simulations, were performed using the Imaris software (Methods). All scale bars, 10 µm.

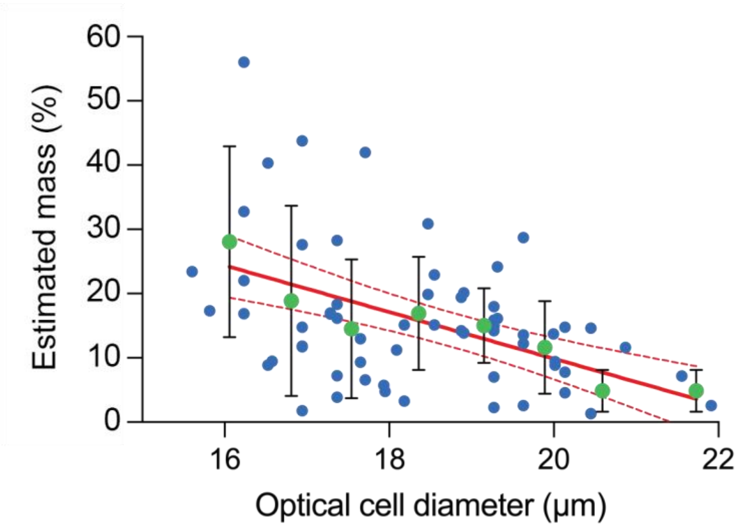


**Supplementary Figure 8. Cell size influences the cell mass measured by oscillating microcantilevers.** For each HeLa cell, the percentage of estimated cell mass is given as derived by dividing the cell mass measured shortly after cell attachment (≤ 1 min) to the microcantilever ($f_{N\_cant} \approx 65 – 90$ kHz) through the volumetric cell mass ($V_{optical} * \rho$) expected from the optically measured cell diameter. The diameter of individual HeLa cells were estimated from their optical images (using Image J, version 2.3.0/1.53q) taken within 1 min after their attachment to the cantilever. The cell density $\rho$ taken was 1.06 g cm$^{-3}$[1]. The graph illustrates that the bigger or heavier the cell, the less accurate the microcantilever-based mass measurement becomes. Individual HeLa cells are shown as blue dots ($n_{\text{cell}}$ = 62). Green dots represent the mean, and error bars indicate the standard deviation after binning the optical diameter into 8 bins. The red solid line represents the linear regression through the mean values and the red dashed lines represent the 95 % confidence interval of the best fit line. A negative correlation of –0.93 and a value of $P$ = 0.0009 were determined by the Pearson correlation test. All measurements were conducted under cell culture conditions. Data taken from Fig. 1b.

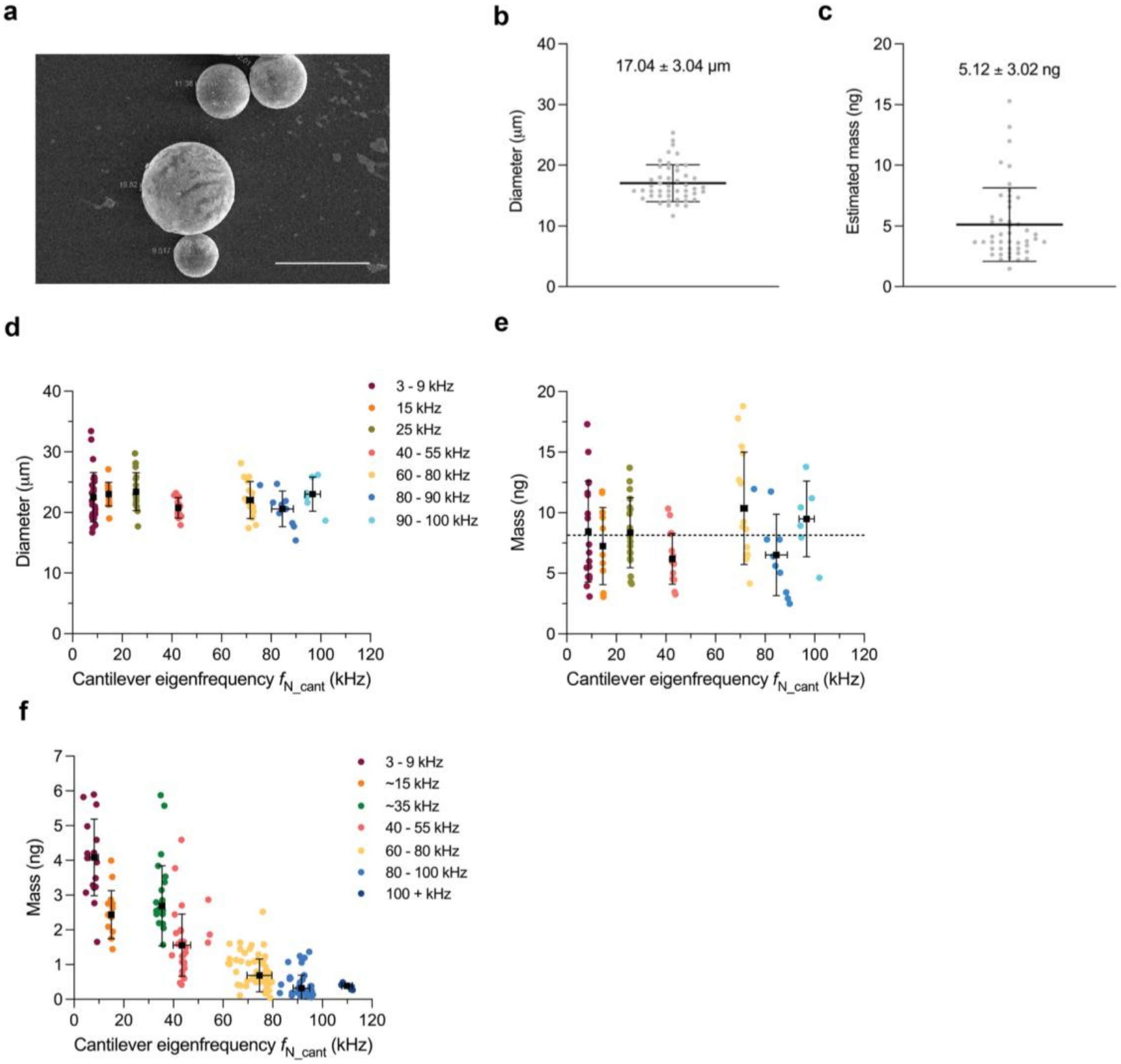


**Supplementary Figure 9. Glass bead characterization and cell mass measurements over a wide eigenfrequency range. a,** Glass beads imaged by scanning electron microscope (SEM). Scale bar, 20 µm. The commercially available glass beads (Kisker-Biotech, PSI-15.0), whose masses have been characterized using oscillating microcantilevers (Fig. 5d), show a large size distribution. **b**, Diameter of glass beads as measured from SEM images. Each dot represents one bead ($n_{\mathrm{bead}} = 48$). Values represent the mean (horizontal black line) and standard deviation (error bars). **c**, Mass of glass beads as derived from the bead diameters measured in **b**. To calculate the bead mass a density of 1.8 g cm$^{-3}$ was used as provided by the manufacturer. Each dot represents the mass of one bead. Values represent the mean (horizontal black line) and standard deviation (error bar). **d**, To prove that glass beads measured over seven different cantilever eigenfrequencies $f_{N\_cant}$ do not largely differ in size, their diameters (corresponding masses are shown in Fig. 5d) were estimated from light microscopy images and Image J software (version 2.3.0/1.53q) (Methods). Each dot represents one bead ($n_{\mathrm{bead}} = 108$), black squares the mean, and error bars the standard deviation. **e**, Total mass of glass beads over seven different cantilever eigenfrequencies $f_{N\_cant}$. Data taken from Fig. 5d. Each colored dot represents a single cell experiment, black squares the mean and error bars the standard deviation ($n_{\mathrm{bead}} = 108$). The dashed black line indicates the mean bead mass of all data points. **f**, Total mass of HeLa cells after attachment (at ≤ 1 min) to ConA-coated microcantilevers over seven different cantilever eigenfrequencies $f_{N\_cant}$. Data taken from Fig. 5b. Each colored dot represents one

single cell experiment, means (black dot) and error bars (standard deviation) are shown in black ($n_{cell}$ = 178).

| | Collagen | | | Matrigel | | | Laminin | | |
|---|---|---|---|---|---|---|---|---|---|
| **Time** | 30 min | 60 min | 120 min | 30 min | 60 min | 120 min | 30 min | 60 min | 120 min |
| **Focal adhesion area (µm$^{-2}$)** | 51 ± 18 | 63 ± 25 | 68 ± 25 | 46 ± 25 | 54 ± 15 | 58 ± 15 | 25 ± 13 | 35 ± 21 | 39 ± 14 |

**Supplementary Table 1. Focal adhesion areas (mean ± SD) of HeLa cells on different substrates and time points after attachment to the substrate.** $n_{cell}$ = 22 – 30 for each substrate and time point. Measurements are taken from Fig. 2b.

| | **30 min** | **60 min** | **120 min** |
|---|---|---|---|
| Collagen vs. matrigel | *ns*, *P*-value: 0.4393 | *ns*, *P*-value: 0.1249 | *ns, P*-value: 0.0604 |
| Collagen vs. laminin | ****, *P*-value: < 0.0001 | ****, *P*-value: < 0.0001 | ****, *P*-value: < 0.0001 |
| Matrigel vs. laminin | ***, *P*-value: 0.0007 | ***, *P*-value: 0.0003 | ****, *P*-value: < 0.0001 |

**Supplementary Table 2. Statistical comparison between focal adhesion areas in Suppl. Table 3 of HeLa cells on different substrates and time points after attachment to the substrate.** Statistical tests were performed using the unpaired t-test with Welch's correction. This two-tailed test was used to test the (null) hypothesis that two populations have equal means. Measurements are taken from Fig. 2b and Supplementary Table 1.

| | Collagen | | | Matrigel | | | Laminin | | | ConA | | |
|---|---|---|---|---|---|---|---|---|---|---|---|---|
| **Time** | 30 min | 60 min | 120 min | 30 min | 60 min | 120 min | 30 min | 60 min | 120 min | 30 min | 60 min | 120 min |
| **Spreading area (µm$^{-2}$)** | 583 ± 175 | 750 ± 197 | 894 ± 316 | 729 ± 300 | 958 ± 231 | 1140 ± 231 | 576 ± 322 | 914 ± 243 | 924 ± 192 | 241 ± 62 | 342 ± 239 | 345 ± 261 |

**Supplementary Table 3. Spreading areas (mean ± SD) of HeLa cells on different substrates and time points after attachment to the substrate.** $n_{cell}$ = 29 – 30 for each substrate and time point. Measurements are taken from Fig. 2c.

| | 30 min | 60 min | 120 min |
|---|---|---|---|
| Collagen vs. matrigel | *, *P*-value: 0.0265 | ***, *P*-value: 0.0005 | **, *P*-value: 0.0029 |
| Collagen vs. laminin | *ns*, *P*-value: 0.9198 | **, *P*-value: 0.0064 | *ns, P*-value: 0.6585 |
| Collagen vs. ConA | ****, *P*-value: < 0.0001 | ****, *P*-value: < 0.0001 | ****, *P*-value: < 0.0001 |
| Matrigel vs. laminin | *ns*, *P*-value: 0.0644 | *ns*, *P*-value: 0.4747 | **, *P*-value: 0.0013 |
| Matrigel vs. ConA | ****, *P*-value: < 0.0001 | ****, *P*-value: < 0.0001 | ****, *P*-value: < 0.0001 |
| Laminin vs. ConA | ****, *P*-value: < 0.0001 | ****, *P*-value: < 0.0001 | ****, *P*-value: < 0.0001 |

**Supplementary Table 4. Statistical comparison between spreading areas in Suppl. Table 1 of HeLa cells on different substrates and time points after attachment to the substrate.** Statistical tests were performed using the unpaired t-test with Welch's correction. This two-tailed test was used to test the (null) hypothesis that two populations have equal means. Measurements are taken from Fig. 2c and Supplementary Table 3.

| Time (min)_cell nr. | Eigenfrequency of the cell (kHz) |
|---|---|
| 5_1 | 15.6 |
| 5_2 | 15.3 |
| 30_1 | 21.3 |
| 30_2 | 21.8 |
| 60_1 | 22.9 |
| 60_2 | 21.5 |

**Supplementary Table 5.** Comsol simulation results for the eigenfrequency of the cell extracted at different time points and different cell spreading geometries (Fig. 3b). The elastic modulus of the cell was chosen to be $E = 1.5$ kPa as found for animal cells[2-4].

**Supplementary Note 1**

To obtain the total cell mass $m_{\mathrm{cell}}$ from the natural resonance frequency of the oscillating microcantilever without and with cell attached, $f_{\mathrm{N\_cant}}$ and $f_{\mathrm{N_{cant}+cell}}$, the measured mass $m_{\mathrm{cell}}^{*}$ (Eq. 1) is corrected by a factor $\frac{1}{\psi(x_{cell})^2}$ (Ref [5,6]):

$$m_{\mathrm{cell}} = m_{\mathrm{cell}}^{*}\frac{1}{\psi(x_{\mathrm{cell}})^2} \qquad \text{Eq. S1}$$

$\psi(x_{\mathrm{cell}})$ describes the mode shape of rectangular cantilevers for their first oscillation mode and is evaluated at the geometric center $x_{\mathrm{cell}}$ (center of mass) of the cell, such that we can write Eq. S2[5]:

$$\psi(x_{\mathrm{cell}}) = \alpha\left(\sin(\xi x_{\mathrm{cell}}) - \sinh(\xi x_{\mathrm{cell}}) + \frac{(\sin(\xi L)+\sinh(\xi L))(\cosh(\xi x_{\mathrm{cell}})-\cos(\xi x_{\mathrm{cell}}))}{\cos(\xi L)+\cosh(\xi L)}\right) \qquad \text{Eq. S2}$$

Here, $\alpha$ is a normalizing constant such that $\psi(L)^2 = 1$ and $L$ being the length of the microcantilever beam.

Thus, after approximating the position of the geometric center (center of mass) of the cell $x_{\mathrm{cell}}$ along the microcantilever beam, this position is used to correct the measured, effective cell mass $m_{\mathrm{cell}}^{*}$ to obtain the total cell mass $m_{\mathrm{cell}}$ (Supplementary Fig. 10 and 11). The dependency of the measured mass on the cell position along the cantilever beam was introduced and explored earlier[5,6]. In general, Eq. 1 and Eq. S1 are a special case of the more general formula presented by Malvar *et al.*[7], where not only the effect of the cell mass, but also the effect of the cell stiffness on the natural resonance frequency shift is discussed. For our application, however, where the soft cell ($10^8$ times lower cell Young's modulus than the microcantilever) is placed at the free end of the microcantilever and where no bending of the microcantilever occurs, only the mass contribution to the natural resonance frequency shift of the cantilever becomes important (see also Supplementary Note 2).

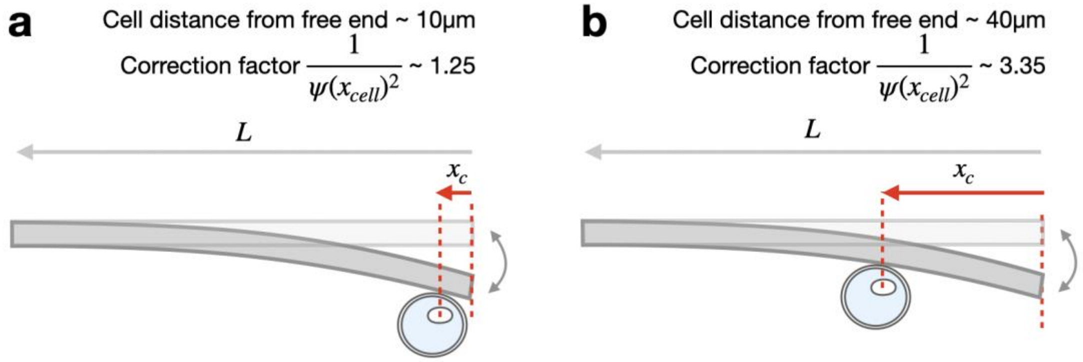


**Supplementary Figure 10. Illustrating the correction of the measured, effective cell mass by the position of the cell along the cantilever beam**. **a**, The rounded cell is positioned ≈ 10 µm from the free end of a $L$ = 120 µm long cantilever beam. This position of gravity of the cell is used to correct the measured, effective mass $m_{\text{cell}}{}^*$ by multiplying by a factor of 1.25. **b**, The rounded cell is positioned ≈ 40 µm from the free end of a $L$ = 120 µm long cantilever beam. This position of gravity of the cell is used to correct the measured, effective mass $m_{\text{cell}}{}^*$ by multiplying by a factor of 3.35.

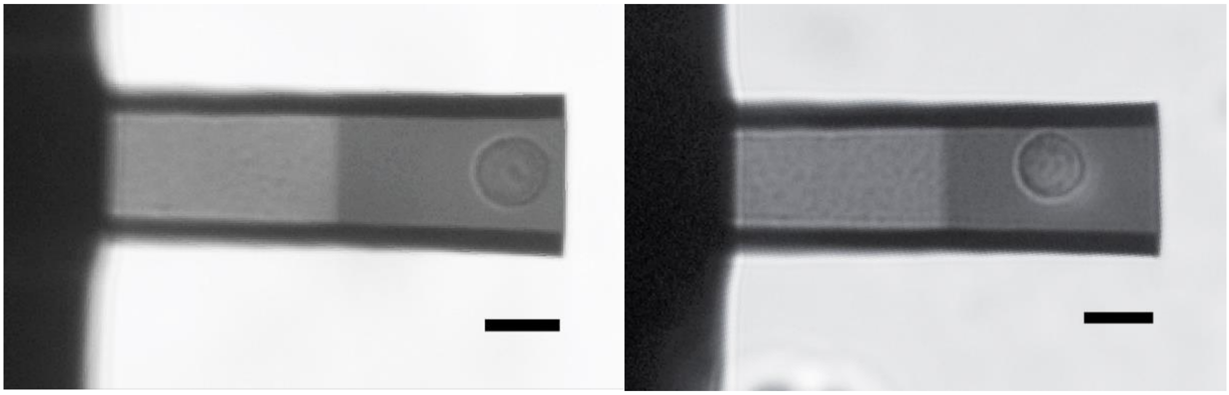

**Supplementary Figure 11. Light microscopy images showing a rounded HeLa cell at different distances along the microcantilever beam**. The microcantilevers shown have a length of $L$ = 120 µm. The different distances of the rounded cells from the free cantilever ends are clearly visible. Scale bars, 10 µm.

**Supplementary Note 2**

To assess the impact of an attached cell on the microcantilever stiffness and subsequently on the natural resonance frequency shift of the cantilever, we applied finite element method (FEM) simulations, which were complemented by experimental controls. The simulations modeled a cell as a hemisphere attached to the free end of a 100 µm long SiN cantilever. By excluding the cell mass in the simulation, we specifically examined the effect of cell stiffness on the natural resonance frequency of the cantilever. We set the cell stiffness to 1 kPa, which is typical for animal cells[2-4], and measured a minimal natural resonance frequency shift of less than 0.01%, which is substantially smaller than the shift of the natural resonance frequency that we typically observe in our experiments upon attachment of a mammalian cell to free end of 840 nm the microcantilever. The simulations also revealed that the first mode shape of the microcantilever, which is the basis of our study, remained unchanged across a wide range of assumed cell stiffnesses, from 1 kPa to 1 GPa (Supplementary Fig. 12a). This constancy suggests that the amount of liquid dragged around by the cantilever (and with it its hydrodynamic function) is independent of the stiffness of the cell. This was not the case for the second mode shape of the cantilever, which we did not use in our experiments.

In addition, we conducted a control experiment to mimic the effect of cell-induced stress without adding mass to the microcantilever. Therefore, we measured the thermal noise spectrum of a 120 µm long microcantilever, both with and without photothermal stress induced by a second laser (840 nm) with static laser power of 1 mW (Supplementary Fig. 12b). The laser was positioned in 15 µm distance from the free end of the cantilever beam. The laser-induced photothermal stress, which was designed to simulate the stress a cell applies upon attachment to the cantilever, deflected the cantilever by ≈ 0.5 nm and caused very minor changes in the microcantilever quality (Q)-factor and the natural resonance frequency. The mechanical stress caused by the static laser beam resulted in an apparent mass increase that was 10 – 100 times smaller than the cell mass.

These findings, both from simulations and experiments, indicate that the natural resonance frequency shift detected upon attachment of a mammalian cell to the free end of the microcantilever is primarily caused by the cell mass rather than through mechanical alterations of the cantilever caused by the cell stiffness or stress (e.g., as mediated by cell adhesion). This negligible role is attributed to the relative softness of the cells, the attachment of the cell to the free end of the cantilever end, and the specific use of the first oscillation mode of the cantilever. All three factors ensure that the cell does not change the mechanical properties of the cantilever in a range that would matter for the outcome of our mass measurements.

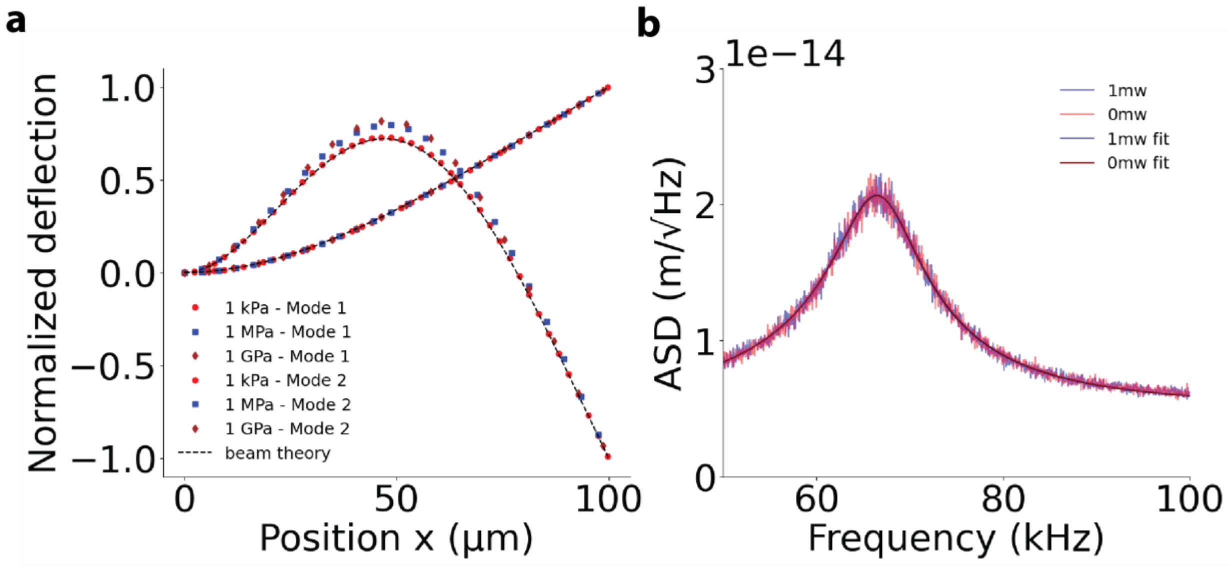


**Supplementary Figure 12. Finite-element simulation and experiments demonstrate that cell stiffness does not affect the cantilever resonance frequency readout in our experiments. a**, Shapes of the first and second mode of a 100 μm long microcantilever beam with cells of varying stiffness attached to it, extracted by finite element method simulations. The cell is placed in the last 20% (80 – 100 μm) of the cantilever. **b**, Natural resonance frequency (eigenfrequency $f_{N_{\text{cant}}}$ and quality ($Q$)-factor of the microcantilever not exposed (0 mW) and exposed (1 mW) to the static laser. Being, not exposed to the static laser the microcantilever shows a natural eigenfrequency $f_{N_{\text{cant}}}$ of 66834 ± 25 Hz and a $Q$-factor of 6.853 ± 0.07. Exposed to the static laser the microcantilever shows a natural eigenfrequency $f_{N_{\text{cant}}}$ of 66785 ± 25 Hz and a $Q$-factor of 6.856 ± 0.03. Experiments were conducted in aqueous solution at room temperature.

## SUPPLEMENTARY REFERENCES


1. Pertoft, H. & Laurent, T. C. in *Methods of Cell Separation* (ed Nicholas Catsimpoolas) 25-65 (Springer US, 1977).
2. Garcia, P. D., Guerrero, C. R. & Garcia, R. Nanorheology of living cells measured by AFM-based force–distance curves. *Nanoscale* **12**, 9133-9143, doi:10.1039/C9NR10316C (2020).
3. Guimarães, C. F., Gasperini, L., Marques, A. P. & Reis, R. L. The stiffness of living tissues and its implications for tissue engineering. *Nat. Rev. Mater.* **5**, 351-370, doi:10.1038/s41578-019-0169-1 (2020).
4. Hayashi, K. & Iwata, M. Stiffness of cancer cells measured with an AFM indentation method. *J. Mech. Behav. Biomed. Mater.* **49**, 105-111, doi:https://doi.org/10.1016/j.jmbbm.2015.04.030 (2015).
5. Martinez-Martin, D. *et al.* Inertial picobalance reveals fast mass fluctuations in mammalian cells. *Nature* **550**, 500-505, doi:10.1038/nature24288 (2017).
6. Incaviglia, I. *et al.* Tailoring the Sensitivity of Microcantilevers To Monitor the Mass of Single Adherent Living Cells. *Nano Lett.* **23**, 588-596, doi:10.1021/acs.nanolett.2c04198 (2023).
7. Malvar, O. *et al.* Mass and stiffness spectrometry of nanoparticles and whole intact bacteria by multimode nanomechanical resonators. *Nat. Commun.* **7**, 13452, doi:10.1038/ncomms13452 (2016).
