# Supplementary material for: Monitoring the mass, eigenfrequency, and quality factor of mammalian cells
Source: Nat Commun. 2024 Feb 26;15:1751. doi: 10.1038/s41467-024-46056-7 (PMC10897412; doi:10.1038/s41467-024-46056-7)
Supplement: Supplementary file 3 — Source Data [file 41467_2024_46056_MOESM3_ESM.pdf]

nature portfolio

Peer Review File

Open Access This file is licensed under a Creative Commons Attribution 4.0 International License, which permits use, sharing, adaptation, distribution and reproduction in any medium or format, as long as you give appropriate credit to the original author(s) and the source, provide a link to the Creative Commons license, and indicate if changes were made. In the cases where the authors are anonymous, such as is the case for the reports of anonymous peer reviewers, author attribution should be to 'Anonymous Referee' followed by a clear attribution to the source work. The images or other third party material in this file are included in the article's Creative Commons license, unless indicated otherwise in a credit line to the material. If material is not included in the article's Creative Commons license and your intended use is not permitted by statutory regulation or exceeds the permitted use, you will need to obtain permission directly from the copyright holder. To view a copy of this license, visit http://creativecommons.org/licenses/by/4.0/.

REVIEWER COMMENTS

Reviewer #1 (Remarks to the Author):

Referee Report on the paper "Monitoring the mass, eigenfrequency, and quality factor of mammalian cells" by S. Herzog et al., submitted to Nature Communications.

In the paper, the authors present extensive and interesting data on the measurements of the resonant frequencies of the AFM cantilevers in liquids, and their changes when a mammalian cell is attached onto the cantilever. The measurements are done with different cantilevers and cells, methods of cell fixations, and at different frequency ranges. The results are interpreted as the measurements of the attached cell mass, eigenfrequency and quality factors.

In my opinion, the following aspect should be clarified before analyzing the Manuscript in further details. This is well known that a cell (as well as cell organells, e.g. liposomes), when attached to an AFM cantilever (to any surface), induces a local stress on its surface, and this stress causes a local deformation of the cantilever. [The effect of the deformation of a substrate due to the stress caused by a deposited foreign layer apparently was first discussed in the paper of G. G. Stoney in 1909. For more modern references, we can indicate e.g. J. Lagowski et al., Appl. Phys. Lett. 26, 493 (1975) or R. Berger et al., Science 276, 2021 (1997) paper; they were followed by hundreds of publications]. Among the effects caused by this stress, there is also the change of the resonance frequency of the plates (cantilevers) due to their (local) deformations (and may be by other reasons); this question also was specially studied, see, among others, aforementioned Lagowski paper or, for example, Lahut and Sader, PRL, 99, 206102 (2007). (Note that the changes made with a cantilever by the local stress depend on the precise localization of the attached cell). The effect is arguably small, right, but the change of this same frequency caused by the added mass (that of the cell) is also small, and both effects should be carefully compared to reliably extract any information from the observed change of the resonance frequency of the system.
For example, in the paper under review, as well as in the Nature 2017 paper of (partly) the same authors, the dependence of the measured cell mass on the attachment chemistry, and rather rapid changes of such a mass are reported. These observations are difficult to understand in the frame of the proposed model. But the dependence of the local stress / deformations of the cantilever on the cell attachment chemistry is absolutely natural, and the possibility of the rapid changes of this same stress for the case of living cells does not look so surprising. Similarly, e.g. the effects of increase of the measured cell mass from 0.5 ng to 2.3 ng as a result of the "chemical crosslinking" (p. 5) is difficult to understand - but this is not at all surprising that such a "chemical crosslinking" can drastically change the adhesion conditions and, as a result, also the stress caused by cell adhesion with all its consequences.
To summarize: we cannot claim that the effect of the AFM cantilever deformation by the stress, induced by attached cell, overwhelms other effects, e.g. the change of the system mass, but it definitely should be carefully studied and assessed.

At a moment, I can recommend a major revision.

Reviewer #2 (Remarks to the Author):

Key results
The manuscript reports about dynamical mass measurements of cells that seem to contradict cell volume and thus mass measurements using the Coulter counter method. The authors provide a sound explanation based on the eigenfrequency of the cells, where the mass of the cell does not count fully when it is mounted on a cantilever that has a higher eigenfrequency than the cells. On the

experimental side, this argument is supported by measurements where the cantilever was loaded with stiff beads.

Overall, I find this report quite interesting and well suited for publication in Nature Communications.

Validity
The crosschecks with glass beads, different cantilever frequencies and theoretical simulations are quite convincing, such that I trust the data and interpretation to be fully valid.

Significance
As my background is atomic force microscopy and quantum nanoscience and my knowledge of biology is quite limited, I do not feel highly qualified to assess the significance of the present study. However, the authors have thoroughly considered the physics of this cell vibrations and I find this approach to bring mechanics into the science of the cell interesting and significant.

Data and methodology
In my view, the validity of the approach, the quality of the data, and the quality of presentation are all very good.

The only thing I missed was a more thorough discussion of the parameter alpha in eq. 1 - perhaps Fig. 1 could contain a little inset with two cell positions and corresponding alpha values.

Analytical approach
... looks fine to me.

Suggested improvements
See above

Clarity and context
The paper was a pleasure to read, regarding context, I do not feel competent to judge because the field is not in my core.

References
Again, I do not feel competent to judge because the field is not in my core.

Your expertise
see above

Monitoring the mass, eigenfrequency, and quality factor of mammalian cells

Point-by-point response to the comments of reviewer #1

Reviewer 1: In the paper, the authors present extensive and interesting data on the measurements of the resonant frequencies of the AFM cantilevers in liquids, and their changes when a mammalian cell is attached onto the cantilever. The measurements are done with different cantilevers and cells, methods of cell fixations, and at different frequency ranges. The results are interpreted as the measurements of the attached cell mass, eigenfrequency and quality factors

In my opinion, the following aspect should be clarified before analyzing the Manuscript in further details. This is well known that a cell (as well as cell organells, e.g. liposomes), when attached to an AFM cantilever (to any surface), induces a local stress on its surface, and this stress causes a local deformation of the cantilever. [The effect of the deformation of a substrate due to the stress caused by a deposited foreign layer apparently was first discussed in the paper of G. G. Stoney in 1909. For more modern references, we can indicate e.g. J. Lagowski et al., Appl. Phys. Lett. 26, 493 (1975) or R. Berger et al., Science 276, 2021 (1997) paper; they were followed by hundreds of publications]. Among the effects caused by this stress, there is also the change of the resonance frequency of the plates (cantilevers) due to their (local) deformations (and may be by other reasons); this question also was specially studied, see, among others, aforementioned Lagowski paper or, for example, Lahut and Sader, PRL, 99, 206102 (2007). (Note that the changes made with a cantilever by the local stress depend on the precise localization of the attached cell). The effect is arguably small, right, but the change of this same frequency caused by the added mass (that of the cell) is also small, and both effects should be carefully compared to reliably extract any information from the observed change of the resonance frequency of the system.

For example, in the paper under review, as well as in the Nature 2017 paper of (partly) the same authors, the dependence of the measured cell mass on the attachment chemistry, and rather rapid changes of such a mass are reported. These observations are difficult to understand in the frame of the proposed model. But the dependence of the local stress / deformations of the cantilever on the cell attachment chemistry is absolutely natural, and the possibility of the rapid changes of this same stress for the case of living cells does not look so surprising. Similarly, e.g. the effects of increase of the measured cell mass from 0.5 ng to 2.3 ng as a result of the “chemical crosslinking” (p. 5) is difficult to understand - but this is not at all surprising that such a “chemical crosslinking” can drastically change the adhesion conditions and, as a result, also the stress caused by cell adhesion with all its consequences.

To summarize: we cannot claim that the effect of the AFM cantilever deformation by the stress, induced by attached cell, overwhelms other effects, e.g. the change of the system mass, but it definitely should be carefully studied and assessed.

Authors: We thank the reviewer for bringing up this important point. Indeed, the change in the natural resonance frequency (eigenfrequency) of the microcantilever $\Delta f_{\mathrm{N\_cant}}$ is affected by changes in the cantilever surface stress s_{cant}. However, below we show that in our experimental setup, this surface stress effect, which is caused by the attached cell, is orders of magnitude smaller compared to the effect of the mass the cell adds to the microcantilever, and even shows the wrong algebraic sign to explain our experimental results. We detail the rationale of this effect the following:

In the 2012 paper from Sader and collegues[1,2], we find an important addition to the 2007 paper from Lachut and Sader[3], which provides an equation for the cantilever's resonance frequency shift caused by the "stress effect" and by the "geometric effect" of the cantilever.
While the stress effect can be described by

$$\frac{\Delta f_{\mathrm{N\_cant}}}{f_{\mathrm{N\_cant}}} = -0.042\nu(b/L)(b/h)^2\bar{\sigma} \qquad \text{(Eq. R1)}$$

with $\bar{\sigma} = (1-\nu)\delta s_{\mathrm{cant}}/Eh$ and L, b, h, E, ν being the length, width, thickness, Young's modulus, and passion ratio of the cantilver, the geometric effect can be described by

$$\frac{\Delta f_{\mathrm{N\_cant}}}{f_{\mathrm{N\_cant}}} = (1+2\nu)(1+\nu)/(2(1-\nu)\bar{\sigma}) \qquad \text{(Eq. R2)}$$

The stress effect considers that surface stress, such as could be applied in our experiments by the adherent cell, stiffens the cantilever and hence increases the spring constant k_{cant} of the cantilever. The geometric effect considers a change in the geometry of the cantilever caused by the surface stress, which typically is not considered because it is small (see also estimations below). For an adherent fibroblast, which roughly shows a diameter of ≈ 20 μm and can generate contraction forces of up to ≈ 20 nN to the surface to which it adheres[4], we can estimate the surface stress of the cantilever to increase by $\delta s_{\mathrm{cant}} \leq -1$ mN m^{-1} (using the negative sign convention for compression stress). Applying the equations provided in reference[1], we find that the magnitude of the stress effect induced relative frequency shift to be $\frac{\Delta f_{\mathrm{N\_cant}}}{f_{\mathrm{N\_cant}}} = 1.1 \times 10^{-10}$ and of the geometric effect induced by the adherent cell to be $\frac{\Delta f_{\mathrm{N\_cant}}}{f_{\mathrm{N\_cant}}} = 3.3 \times 10^{-9}$. Both values are orders of magnitude smallers than the few percent of frequency shift $\frac{\Delta f_{\mathrm{N\_cant}}}{f_{\mathrm{N\_cant}}} \approx 10^{-2}$ we observe upon attaching a cell to the free end of the microcantilever. To further theoretically consider the relative impact of the mass added by the cell to the microcantilever and the surface stress applied by the cell to the microcantilever in an liquid environment, we considered reference[5]. According to reference[5], we can write $\delta k_{\mathrm{cant}} = \frac{\pi^2}{4n_1}\delta s_{\mathrm{cant}}$ (Eq. R3) with n_1 = 0.24[5]. The resonance frequency shift of the microcantilever as induced by the cell mass added and the surface stress applied by the cell follows: $\Delta f_{\mathrm{N\_cant}} = \frac{f_{\mathrm{N\_cant}}}{2}\left(\frac{\delta k_{\mathrm{cant}}}{k_{\mathrm{cant}}} - \frac{m_{\mathrm{cell}}}{m_{\mathrm{cant}}}\right)$ (Eq. R4), where m_{cant} is the cantilever mass, which in our case is an effective mass accounting for the liquid dragged around by the cantilever, and m_{cell} the mass of the cell attached to the cantilever.

For typical values in our study (k_{cant} = 10 N m^{-1}, m_{cant} = 80 ng, m_{cell} = 3 ng) we find $\frac{\delta k_{\mathrm{cant}}}{k_{\mathrm{cant}}}$ = 0.001 and $\frac{m_{\mathrm{cell}}}{m_{\mathrm{cant}}}$ = 0.0375. Hence, in this estimation, the contribution to the natural resonance frequency shift of the microcantilever caused by the added cell mass is 37.5 times bigger than the frequency shift caused by the cell stress, and hence about 100 times bigger than the geometric effect. It is important to note that here we overestimated $\frac{\delta k_{\mathrm{cant}}}{k_{\mathrm{cant}}}$ considerably as Eq. R4 implies that the surface stress applied by the cell δs_{cant} acts across the entire microcantilever, whereas in reality the cell only resides on only 8 – 25% of the cantilever length (see for example **Fig. 1** in which the cell locates at the end of the microcantilever only). That the cell only partly covers the cantilever becomes particulary important for the longest (*i.e.*, heaviest) and

softest microcantilevers used in our study (**revised Table 1**), for which one could expect the relative change of the cantilever spring constant $\frac{\delta k_{\text{cant}}}{k_{\text{cant}}}$ to become biggest (due to small k_{cant}) and of the mass $\frac{m_{\text{cell}}}{m_{\text{cant}}}$ to become smallest (due to a big m_{cant}). Using finite element simulations (**Fig. R1a**), however, we find that the contribution of the microcantilever surface stress applied by the cell δs_{cant} to the natural resonance frequency shift $\Delta f_{\text{N\_cant}}$ can be safely neglected compared to the cell mass effect (see **Fig. R1b**). Intuitively, the surface stress caused by the adherent cell is not important for our cell mass measurements, as the microcantilevers used are relatively stiff, the cell exerts little force (*e.g*., tension) compared to the large cell mass, and the cell resides at the free end of the cantilever, where almost no bending of the cantilever occurs. Hence, the cell induced surface stress does not contribute to an increased bending rigidity (see **Fig. R1b**).

Lastly, our experiments also point out that $\frac{\delta k_{\text{cant}}}{k_{\text{cant}}}$ is negligible (**Fig. 5**). Upon chemical fixation of the indvidual cells attached to the free end of the microcantilever, we experimentally observe either a constant mass (**Fig. 5f**), and hence $\Delta f_{\text{N\_cant}} \approx 0$, or a mass increase (**Figs. 2d** and **5e**), and hence $\Delta f_{\text{N\_cant}} << 0$. As revealed from Eq. R3, a positive $\frac{\delta k_{\text{cant}}}{k_{\text{cant}}}$, such as caused by chemically fixation of the cell, which increases the cell stiffness, an increasing surface stress, or adhesion of the cell to the microcantilever, should shift the natural resonance frequency of the microcantilever to positive vaues ($\Delta f_{\text{N\_cant}} > 0$). As a consequence the mass of the fixed cell as detected by the resonating microcantilever would decrease. However, in our mass measurements we do not observe the cell mass to decrease after chemical fixation (**Fig. 5e,f**), neither we observe a mass decrease while the cells increase adhesion (**Fig. 2d,e**).

In summary, the theoretical considerations, simulations and experimental results allow to conclude that possible effects of the cell applying surface stress of the microcantilever can safely be ignored in our experimental setup, as this effect is too small compared to that of the mass increase and, in addition, has the wrong algebraic sign to explain our experimental results. At the same time, our results can be easily explained in our proposed framework and experimental testing.

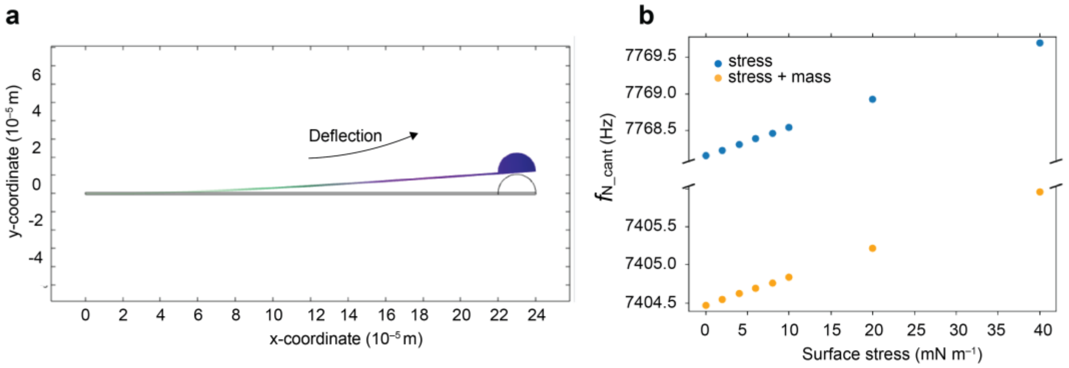

Figure R1. Finite element simulations showing that the surface stress induced spring constant change and shift of the natural resonance frequency of the microcantilever by the adherent cell are negligible compared to the changes caused by the mass added by the adherent cell. a, COMSOL model and mode shape of the microcantilever with attached cell. Black and white are the original undeflected cantilever and cell geometry. The colorized deflected cantilever geometry shows that the bending of the cantilever predominantly occurs in the region of changing color (i.e., towards the cantilever center) and not at the region of the cell attachment. **B**, Change in the natural resonance frequency of the cantilever as a function

of the surface stress applied and the mass of 5.5 ng added by an adhering cell. The cell parameters assumed for this simulation were those of a typical fibroblast in the adherent state having a diameter of ≈ 20 µm and applying a cantilever surface stress increase δs_{cant} of up to 20 mN m^{-1}.

Monitoring the mass, eigenfrequency, and quality factor of mammalian cells

Point-by-point response to the comments of reviewer #2

Reviewer 2: The manuscript reports about dynamical mass measurements of cells that seem to contradict cell volume and thus mass measurements using the Coulter counter method. The authors provide a sound explanation based on the eigenfrequency of the cells, where the mass of the cell does not count fully when it is mounted on a cantilever that has a higher eigenfrequency than the cells. On the experimental side, this argument is supported by measurements where the cantilever was loaded with stiff beads.
Overall, I find this report quite interesting and well suited for publication in Nature Communications.

Authors: Thank you for your encouraging feedback and for the constructive criticism and comments. Below we list point-by-point how we addressed each specific concern of the reviewer.

Reviewer 2: The crosschecks with glass beads, different cantilever frequencies and theoretical simulations are quite convincing, such that I trust the data and interpretation to be fully valid.

Authors: Thank you.

Reviewer 2: As my background is atomic force microscopy and quantum nanoscience and my knowledge of biology is quite limited, I do not feel highly qualified to assess the significance of the present study. However, the authors have thoroughly considered the physics of this cell vibrations and I find this approach to bring mechanics into the science of the cell interesting and significant.

Authors: Thank you very much for your encouraging comment.

Reviewer 2: In my view, the validity of the approach, the quality of the data, and the quality of presentation are all very good.

Authors: Thank you again.

Reviewer 2: The only thing I missed was a more thorough discussion of the parameter alpha in eq. 1 - perhaps Fig. 1 could contain a little inset with two cell positions and corresponding alpha values.

Authors: Thank you. We apologize for the confusion created. We now use a less confusing denomination and properly introduce the correction factor in our revised manuscript. In our initially submitted manuscript we had described the cell mass m_{cell} as:

$$m_{\text{cell}} = \alpha \frac{k_{\text{cant}}}{4\pi^2}\left(\frac{1}{f^2_{\text{N\_cant+cell}}} - \frac{1}{f^2_{\text{N\_cant}}}\right) \qquad \text{former Eq. 1 of manuscript}$$

To obtain the total cell mass from the natural resonance frequency (eigenfrequency) shift of the oscillating microcantilever, the measured, apparent mass $m_{\text{cell}}{}^*$ has to be multiplied by a factor α, which corrects the mass for the cell position sensitivity of the

measurement (Fig. R3).[6] However, the correction for the cell position was denominated differently in previous publications[6,7]. In addition, we realize that the factor α had been already used as a normalizing constant in the equation to describe the mode shape equation of rectangular cantilevers $\psi(x_{\text{cell}})$ for the first oscillation mode of the cantilever (Eq. R5)[6,7], hence the confusion.

$$\psi(x_{\text{cell}}) = \alpha\left(\sin(\xi x_{\text{cell}}) - \sinh(\xi x_{\text{cell}}) + \frac{(\sin(\xi L)+\sinh(\xi L))(\cosh(\xi x_{\text{cell}})-\cos(\xi x_{\text{cell}}))}{\cos(\xi L)+\cosh(\xi L)}\right) \quad \text{Eq. R5}$$

In our revised manuscript, we now use a consistent nomenclature where α is a normalizing constant such that $\psi(L)^2 = 1$ and L the total length of the microcantilever. The frequency shift induced by the attached mass depends on the position of the mass along the cantilever. Thus, the detected cell mass is proportional to the square of the cantilever mode shape amplitude $\psi(x_{\text{cell}})$ evaluated at the cell position x_{cell}, such that the total mass of the cell m_{cell} can be calculated as:[6,7]

$$m_{\text{cell}} = m_{\text{cell}}^* \frac{1}{\psi(x_{\text{cell}})^2} \qquad \text{Eq. R6}$$

where m_{cell}^* is the apparent mass of the cell, which is calculated from the natural resonance frequency shift as follows:

$$m_{\text{cell}}^* = \frac{k_{\text{cant}}}{4\pi^2}\left(\frac{1}{f^2_{\text{N\_cant+cell}}} - \frac{1}{f^2_{\text{N\_cant}}}\right) \qquad \text{revised Eq. 1 of manuscript}$$

To remove any confusion of the reader we have corrected our manuscript and now simply use the same denomination as in previous publications.[2,6,7] In addition, in our revised manuscript we now clearly state that m_{cell}^* has to be corrected using the mode shape equation of the cantilever $\psi(x_{\text{cell}})$, evaluated at the postion of the cell x_{cell} along the cantilever beam (Eq. R5 and R6). We have now included a **Supplementary Note 1** and **Supplementary Figs. S10** and **S11** into our revision to explain this issue at better detail. As suggested by the reviewer, the supplementary figures exemplify two cells having different positions x_{cell} along the cantilever and thus needing different correction factors to provide the correct cell mass (following Eq. R6).

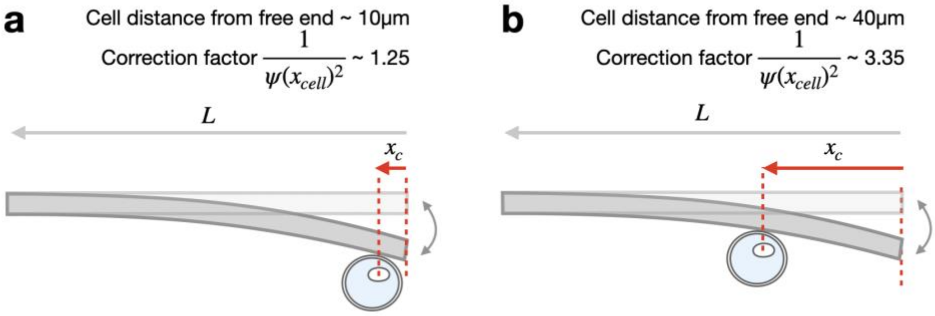

Fig. R3, included into the revised manuscript as new Supplementary Figure S10. Illustrating the correction of the apparent cell mass by the position of the cell along the cantilever beam. **a**, The rounded cell is positioned ≈ 10 µm from the free end of a L = 120 µm long cantilever beam. This center of mass position of the cell is used to correct the apparent mass m_{cell}^* by multiplying by a factor of 1.25. **b**, The rounded cell is positioned ≈ 40 µm from the free end of a L = 120 µm long cantilever beam. This center of mass position of the cell is used to correct the apparent mass m_{cell}^* by multiplying by a factor of 3.35.

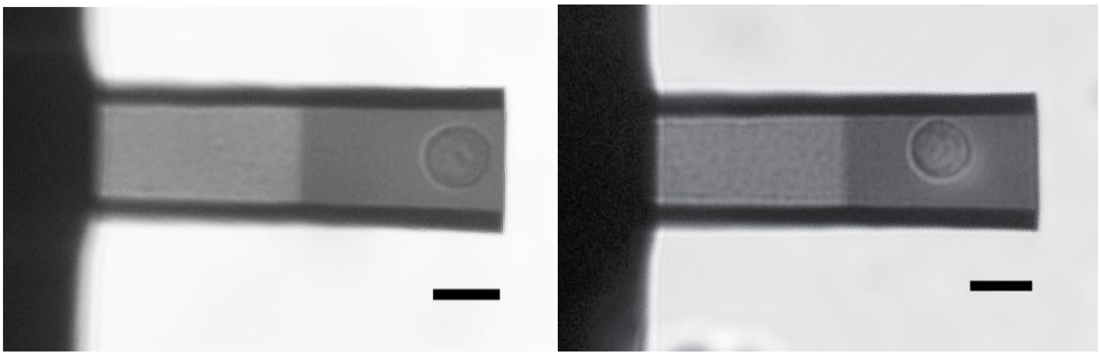

Fig. R4, included into the revised manuscript as new Supplementary Figure S11. Light microscopy images showing a rounded fibroblast at different distances along the microcantilever beam. The microcantilever beams have lengths of L = 120 µm. The different positions of the rounded cells from the free cantilever ends are clearly visible. Scale bars, 10 µm.

References

1 Karabalin, R. B., Villanueva, L. G., Matheny, M. H., Sader, J. E. & Roukes, M. L. Stress-Induced Variations in the Stiffness of Micro- and Nanocantilever Beams. *Phys. Rev. Lett.* **108**, doi:10.1103/PhysRevLett.108.236101 (2012).

2 Cuny, A. P. *et al.* High-resolution mass measurements of single budding yeast reveal linear growth segments. *Nat. Commun.* **13**, 3483, doi:10.1038/s41467-022-30781-y (2022).

3 Lachut, M. J. & Sader, J. E. Effect of surface stress on the stiffness of cantilever plates. *Phys. Rev. Lett.* **99**, 206102, doi:10.1103/PhysRevLett.99.206102 (2007).

4 Balaban, N. Q. *et al.* Force and focal adhesion assembly: a close relationship studied using elastic micropatterned substrates. *Nat. Cell Biol.* **3**, 466-472, doi:10.1038/35074532 (2001).

5 Chen, G. Y., Thundat, T., Wachter, E. A. & Warmack, R. J. Adsorption-Induced Surface Stress and Its Effects on Resonance Frequency of Microcantilevers. *J. Appl. Phys.* **77**, 3618-3622, doi:Doi 10.1063/1.359562 (1995).

6 Martinez-Martin, D. *et al.* Inertial picobalance reveals fast mass fluctuations in mammalian cells. *Nature* **550**, 500-505, doi:10.1038/nature24288 (2017).

7 Incaviglia, I. *et al.* Tailoring the Sensitivity of Microcantilevers To Monitor the Mass of Single Adherent Living Cells. *Nano Lett.* **23**, 588-596, doi:10.1021/acs.nanolett.2c04198 (2023).

REVIEWER COMMENTS

Reviewer #2 (Remarks to the Author):

I was already very pleased with the first version of the manuscript. In my view, the authors have addressed the concerns of the other reviewers satisfactorly and the manuscript is fit for publication.

Reviewer #3 (Remarks to the Author):

The coupling of mass and stiffness effects in the resonant frequency shift of the cantilever is very subtle. In general, the effects associated with the stiffness arise from the moment of inertial of the cantilever, which depends on its geometry and elastic properties. When the particle size is considerable smaller than the cantilever size, the stiffness effects might be ignored. However, the situation might change if the particle thickness (cell's height) is comparable to the cantilever thickness. The core of the authors response lies on some theoretical approximations which separate mass and stiffness effects. Those approximations might be out of date. I would suggest to reanalyze the data in view of the model developed by Tamayo's group (Nat. Commun. 7, 13452 (2016) and references therein).

Reviewer #1 (Remarks to the Author):

In my previous Report, I, after admitting certain interest and importance of the paper, put the question that the effect of the change of the shape and other parameters of the cantilever, following the adsorption of cells onto them, on their resonant frequencies must be carefully estimated. Unfortunately, after assessing the revised version of the Manuscript and authors' reply, I do not feel that this question has been satisfactorily addressed.

The authors now present some estimations based mainly on the results of the paper of R. B. Karabalin *et al.*, PRL 108, 236101 (2012) to claim that the effect of the change in shape and other parameters of the cantilever, caused by cell adsorption, on its resonant frequency is very small and might be neglected. However, this paper is not very relevant for the question. (In my previous review, the references were given only to indicate the principles and not for calculations/estimations concerning the actual experimental conditions). In this paper, R. B. Karabalin *et al.* discuss the resonant frequency of the cantilevers *in vacuum* (air) while the experiments in the Manuscript under review are done *in liquids*. The relation between the resonant frequency of the cantilever in vacuum and in liquid is complicated (see e.g. the paper by Green and Sader *J. Appl. Phys.* 98, 114913 (2005) referenced in the Manuscript under review as Ref. 81, or many references therein, or elsewhere), and the results obtained in vacuum cannot be directly applied to the dithering in liquids. The main factor influencing the resonant frequency in liquid is the so-called *hydrodynamic function* for oscillations, which describes the "viscous" involvement of huge quantity of liquid to the dithering of the lever. To cite the aforementioned *J. Appl. Phys.* Paper about this function: "essentially the normalized hydrodynamic loading of an infinitely long rigid beam of identical cross section to the cantilever beam under consideration", "this… function *is dependent upon the cross section of the beam* [italic mine], and is found by solving the governing unsteady Stokes equations". This hydrodynamic function is rather sensitive to the cantilever shape modified by the cell adsorption or adhesion. I can only repeat from my previous Report: the question putting therein should be very seriously investigated.

Finally, and just for the completeness: the formula Eq. R2 $\frac{\Delta f_{N,cant}}{f_{N,cant}} = \frac{(1+2\nu)(1+\nu)}{2(1-\nu)}\bar{\sigma}$ (written by the authors as $\frac{\Delta f_{N,cant}}{f_{N,cant}} = (1+2\nu)(1+\nu)/(2(1-\nu)\bar{\sigma})$, that is $\frac{\Delta f_{N,cant}}{f_{N,cant}} = \frac{(1+2\nu)(1+\nu)}{2(1-\nu)\bar{\sigma}}$) describes the geometric effect (in vacuum) for the case of *doubly clamped beam* and not for the case of cantilever beam, where the

formula is $\frac{\Delta f_{N,cant}}{f_{N,cant}} = \frac{1+2\nu}{1-\nu}\bar{\sigma}$. (Of course, the difference is only a factor of 2, but still).

I recommend rejection.

Monitoring the mass, eigenfrequency, and quality factor of mammalian cells

Point-by-point response to the comments of reviewer #1

Reviewer: In my previous Report, I, after admitting certain interest and importance of the paper, put the question that the effect of the change of the shape and other parameters of the cantilever, following the adsorption of cells onto them, on their resonant frequencies must be carefully estimated. Unfortunately, after assessing the revised version of the Manuscript and authors' reply, I do not feel that this question has been satisfactorily addressed.

The authors now present some estimations based mainly on the results of the paper of R. B. Karabalin et al., PRL 108, 236101 (2012) to claim that the effect of the change in shape and other parameters of the cantilever, caused by cell adsorption, on its resonant frequency is very small and might be neglected. However, this paper is not very relevant for the question. (In my previous review, the references were given only to indicate the principles and not for calculations/estimations concerning the actual experimental conditions). In this paper, R. B. Karabalin et al. discuss the resonant frequency of the cantilevers in vacuum (air) while the experiments in the Manuscript under review are done in liquids. The relation between the resonant frequency of the cantilever in vacuum and in liquid is complicated (see e.g. the paper by Green and Sader J. Appl. Phys. 98, 114913 (2005) referenced in the Manuscript under review as Ref. 81, or many references therein, or elsewhere), and the results obtained in vacuum cannot be directly applied to the dithering in liquids. The main factor influencing the resonant frequency in liquid is the so-called hydrodynamic function for oscillations, which describes the "viscous" involvement of huge quantity of liquid to the dithering of the lever. To cite the aforementioned J. Appl. Phys. Paper about this function: "essentially the normalized hydrodynamic loading of an infinitely long rigid beam of identical cross section to the cantilever beam under consideration", "this…function is dependent upon the cross section of the beam [italic mine], and is found by solving the governing unsteady Stokes equations". This hydrodynamic function is rather sensitive to the cantilever shape modified by the cell adsorption or adhesion. I can only repeat from my previous Report: the question putting therein should be very seriously investigated.

Finally, and just for the completeness: the formula Eq. R2, $\frac{\Delta f_{N,cant}}{f_{N,cant}} = \frac{(1+2\nu)(1+\nu)}{2(1-\nu)}\bar{\sigma}$ (written by the authors as $\frac{\Delta f_{N,cant}}{f_{N,cant}} = (1+2\nu)(1+\nu)/(2(1-\nu)\bar{\sigma})$, that is $\frac{\Delta f_{N,cant}}{f_{N,cant}} = \frac{(1+2\nu)(1+\nu)}{2(1-\nu)\bar{\sigma}}$) describes the geometric effect (in vacuum) for the case of doubly clamped beam and not for the case of cantilever beam, where the formula is $\frac{\Delta f_{N,cant}}{f_{N,cant}} = \frac{1+2\nu}{1-\nu}\bar{\sigma}$. (Of course, the difference is only a factor of 2, but still).

I recommend rejection.

Authors: We thank the reviewer for the continued effort, although we are somewhat surprised by the response, particularly, in the light of the first round of reviews.

In the first review reviewer 1 writes: “To summarize: we cannot claim that the effect of the AFM cantilever *deformation by the stress* [italic by us], induced by attached cell, overwhelms other effects, e.g. the change of the system mass, but it definitely should be carefully studied and assessed.“

In our corresponding response, we had addressed the reviewer’s concern about the possible cantilever *deformation by cell induced stress*, showing that little influence is to be expected. Yet, in the second review the reviewer asks a different question, in which cell induced stress is not mentioned anymore, claiming that at the same time we would not have satisfactorily answered the concern the reviewer has made in the first review. The current question of the reviewer is phrased in the context of the hydrodynamic function of the cantilever and the effect of the cell geometry on the cross section of the cantilever. We will also address this issue as best as possible, however, reserving a more comprehensive answer for a dedicated research project which demands the complexity of the question. Following the detailed experiments, simulations, and controls presented in our manuscript, it is not entirely clear why and how the hydrodynamic function – despite being an interesting topic – could be relevant to our reported insights, as we will address in the following:

1) Our research paper mainly focusses on measuring cell mass using microcantilevers with different natural resonance frequencies (eigenfrequencies). The cells attached to the microcantilevers have very similar shapes as they are all from the same cell line and cell culture, and have been freshly attached to the substrates coating the microcantilever. Even minutes after attachment to the microcantilever, the cells are mainly round and very similar in shape. Yet, the experimental results of our measurements vary considerably, depending on the natural resonance frequency of the microcantilever. Thus, this effect is unlikely to be explained by a different hydrodynamic function, since all microcantilevers used in our study are rectangular beams, for which Sader provided the hydrodynamic function, and the cells have similar shapes. Therefore, we expect the hydrodynamic function to be very similar for the different experimental conditions tested.
2) To further support our point, we conducted finite element method (FEM) simulations (Figure R1). These simulations involve a rod in water flow, representing the cross section of a simplified cantilever. When two differently shaped adsorbates were added, which would represent cell shape changes, the drag force changed compared to the non-absorbed case, but the force did not depend on the different shape of the adsorbate (cells). This finding underscores that the differential measurements performed in our study are reasonable.
3) The indifference of FEM simulations to reasonable changes in cell shape is in line with analytical results concerning the hydrodynamic force on spherical shapes and deviations of such.[1] The intuitive understanding here is that the shape of the absorbate only plays a role in the integral formulation. An in-depth understanding of the exact geometries requires either sophisticated approaches to solving the Navier-Stokes analytically, which seems unlikely given the already existing treatments and the lack of such solution, or an extensive numerical treatment, which is a research project of its own.
4) In our chemical fixation experiments (Figure 2d and 5e,f), we did not change the shape of the cell and, therefore, the cross section of the cantilever-cell system. In the first chemical fixation experiments using high eigenfrequency microcantilevers, we show that not cell shape (which does not change) but cell stiffening recovers the proper cell mass measurements (Figure 2d). In addition, the discovery that low eigenfrequency microcantilevers measure the accurate cell mass as measured with cantilevers of chemically fixed cells shows that our measurements are independent of any effect of the hydrodynamic function (Figure 5e,f). Both findings also nicely underscore the

unique potential of the differential cell mass measurements (*e.g.*, comparing two experiments by varying only one experimental parameter), and allow to draw the conclusions presented in our work.

5) A change of the hydrodynamic function is very unlikely to explain our results. The hydrodynamic function, which describes the 'normalized hydrodynamic load', depends on the frequency. In fact, as the cantilever eigenfrequency increases, so should the hydrodynamic load as it is proportional to the velocity of the cantilever movement. Yet, at higher frequencies we observe smaller cell masses. However, we can again explain this startling effect using our proposed theoretical framework.

In summary, we agree with the reviewer that an altered hydrodynamic function might be a source of some uncertainty for the absolute measurement, which we have therefore added as a consideration in the discussion. Our results, however, show that the effect does not jeopardize the results and findings made in our manuscript.

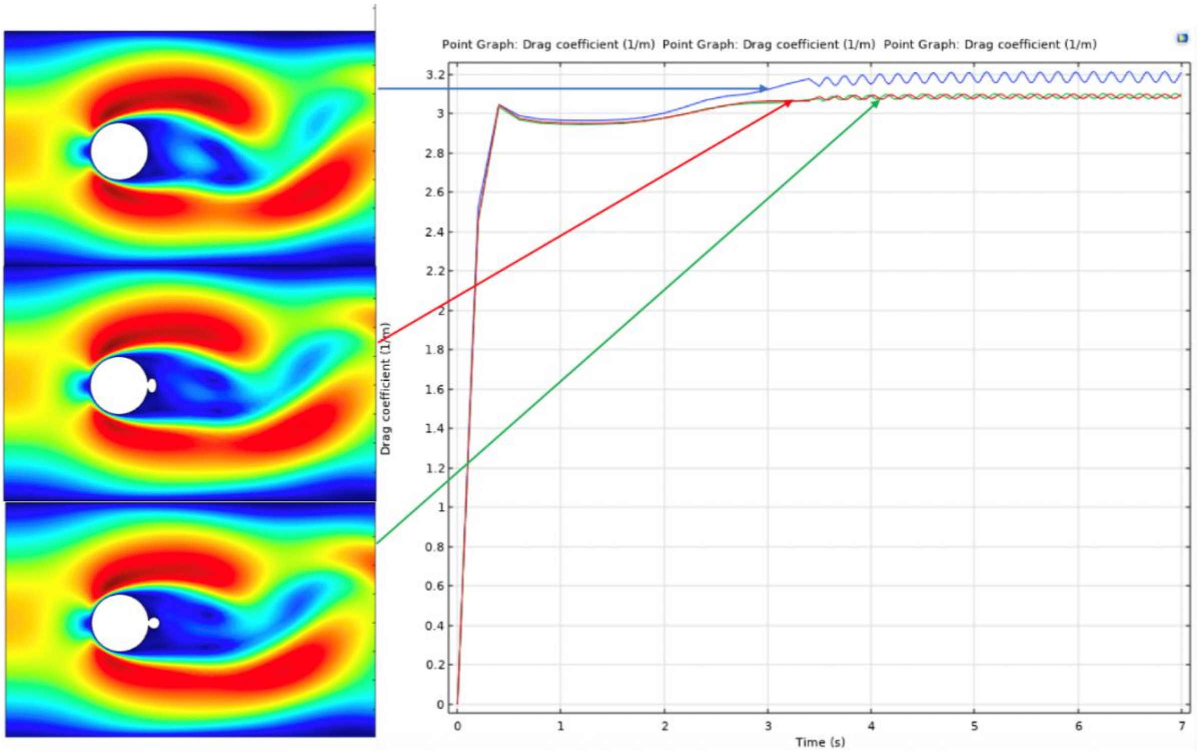

Figure R1. 2D Finite element method (FEM) simulations demonstrating the effect on an absorbate changing the cross-sectional area of a structuring interacting with a fluid. Left top, Cross section (xy-plane) of an idealized cantilever without adsorbate (white) immersed in a fluid (velocity field depicted from blue (slow) to red (fast)). The long z-axis of the cantilever is not shown in the projection. Left middle and bottom, the same cantilever and with differently shaped absorbates (white), in a fluid (velocity field depicted from blue (slow) to red (fast)). Right, corresponding drag coefficient over time, calculated from the FEM simulation. In the beginning the flow is switched on. After about 3.5 s the system reaches a stationary behavior.

Reference

1. Zhang, W., & Stone, H. (1998). Oscillatory motions of circular disks and nearly spherical particles in viscous flows. *J Fluid Mech, 367*, 329-358. doi:10.1017/S0022112098001670

Monitoring the mass, eigenfrequency, and quality factor of mammalian cells

Point-by-point response to the comments of reviewer #2

Reviewer: I was already very pleased with the first version of the manuscript. In my view, the authors have addressed the concerns of the other reviewers satisfactorly and the manuscript is fit for publication.

Authors: Thank you very much for your positive and constructive review, which guided us to further improve our paper.

Monitoring the mass, eigenfrequency, and quality factor of mammalian cells

Point-by-point response to the comments of reviewer #3

Reviewer: The coupling of mass and stiffness effects in the resonant frequency shift of the cantilever is very subtle. In general, the effects associated with the stiffness arise from the moment of inertial of the cantilever, which depends on its geometry and elastic properties. When the particle size is considerable smaller than the cantilever size, the stiffness effects might be ignored. However, the situation might change if the particle thickness (cell's height) is comparable to the cantilever thickness. The core of the authors response lies on some theoretical approximations which separate mass and stiffness effects. Those approximations might be out of date. I would suggest to reanalyze the data in view of the model developed by Tamayo's group (Nat. Commun. 7, 13452 (2016) and references therein).

Authors: Thank you for your constructive suggestion on how to test the validity of our model, particularly on '*the effects associated with the stiffness arise from the moment of inertial of the cantilever, which depends on its geometry and elastic properties*'. For testing the validity of our model, this reviewer suggested reanalyzing our experimental data in view of the model developed by the Tamayo's group[1]. In light of the suggestions, we reanalyzed the experimental data shown in Figure 5f, which shows the mass measurements of living and crosslinked HeLa cells adhering to low resonance frequency cantilevers (Figure R2). Because crosslinking considerably changes the stiffness of HeLa cells (Supplementary Fig. 6), the effects associated with stiffness changes should become particularly prominent for this experiment, additionally also as the cantilever type used had the lowest spring constant of all the used cantilevers. As this reanalysis of the experimental data shows (Figure R2), however, the model applied by the Tamayo group (Malvar *et al.* Nat. Commun. 7, 13452 (2016)[1] and references therein) does not change the readout of the total mass of HeLa cells.

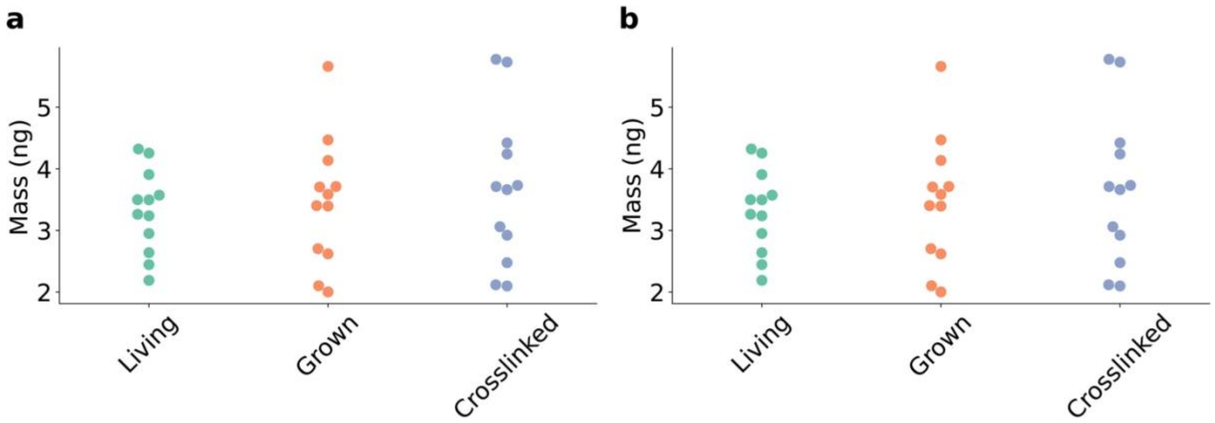

Figure R2. Analysis of the total mass of living and crosslinked HeLa cells adhering to low resonance frequency microcantilevers using the methods provided by (a) Martinez-Martin *et al.* (2017) [2] and (b) Malvar *et al.* (2016[1]. The two different analyses of the experimental data recorded in mass measurements do not result in significantly different mass values. For experimental data taken for analysis has been taken from Figure 5f.

The reason for not finding any different results using the model from the Tamayo group, can be identified when examining the different contributions in the stiffness terms presented by Malvar *et al.* (2016)[1]:

$$\Omega_m = 1/2\lambda_m(-\psi_n^2(x_0) + 3 * C_s * \kappa_n^2(x_0)) \quad \text{(Eq. R1)}$$

with Ω_m being the fractional shift of the cantilevers resonance frequency, λ_m the mass ratio of absorbate (in our case the cell and cantilever), ψ_n^2 the square of the mode shape, C_s the absorbate stiffness factor, and κ_n^2 the square of the normalized curvature of the mode shape of the microcantilever.

Curvature of the mode shape of the microcantilever: Figure R3 displays the square of the normalized curvature mode shape of the microcantilever κ_n^2, where x is the normalized position on the cantilever. At x_0 = 0.8 the square curvature is merely 0.017, and even lower for positions closer to the free end of the microcantilever at which we usually positioned the HeLa cell for mass measurements (Figure 1). Here, we find the first indication that the stiffness effect does not play a role in changing the resonance frequency of the microcantilever. Furthermore, Malvar *et al.* write '*the stiffness effect increases in the regions where the beam undergoes higher changes of curvature during vibration (higher potential energy)*'.[1] In our case, however, the HeLa cell locates close to the free end of the microcantilever, where no bending occurs in the first mode shape of the microcantilever.

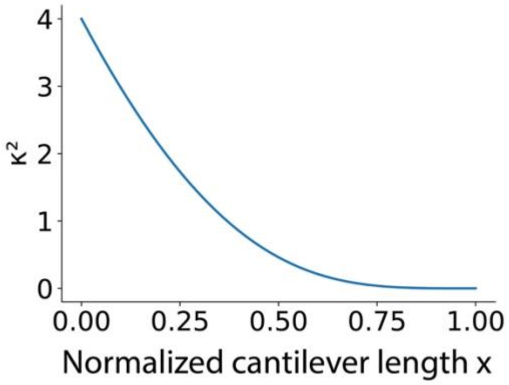

Figure R3. Normalized second derivative of mode shape (normalized curvature) of the microcantilever. κ_n^2 is the square of the normalized curvature of the mode shape of the microcantilever and x is the normalized length of the microcantilever.

Absorbate stiffness: The next term of Eq. R1 is the stiffness absorbate factor C_s, which is calculated as $C_s = \varepsilon * \left(\frac{E_a}{E_b}\right)\left(\frac{\rho_b}{\rho_a}\right)$, where E_a and E_b are the Young's moduli of the absorbate and the microcantilever beam, respectively. ρ_a and ρ_b are the densities of absorbate and microcantilever, and ε is a factor describing the geometric influence of the absorbate to the flexural rigidity of the microcantilever. The ratio $\left(\frac{\rho_b}{\rho_a}\right)$ is 2.2. The ratio $\left(\frac{E_a}{E_b}\right)$ is $2.4 \cdot 10^{-8}$ assuming a Young's modulus of 150 GPa for silicon[3], whereas the HeLa cell only has a Young's modulus of ≈ 2 kPa,[4] which is more than 8 decades (10^8) softer than the microcantilever. Next to the inexistent bending at the end of the cantilever (see above), this is the second important indication why the stiffness effect does not play a role in our mass measurements. This situation described for our mass measurements is in contrast to the measurements presented by the Tamayo group[1], since the stiffness of air-dried bacteria of 1 GPa is ≈ 10^6 times stiffer

than the stiffness of HeLa cells and, therefore, lies much closer to the Young's modulus of the silicon cantilever of 150 GPa.

Geometric influence: What remains to be considered is the ε factor. For making the stiffness effect on the resonance frequency shift comparable to the mass effect it would need to be in the order of ≈10^{10}, as $\kappa_n^{2}*\left(\frac{E_a}{E_b}\right)\left(\frac{\rho_b}{\rho_a}\right)$ (see Eq. R1) is ≈ 10^{-10}. At the same time, the mass contribution to the resonance frequency shift is of the order of 1, as can be found be evaluating $-\psi_n^2(x_0)$ (see Eq. R1). Malvar *et. al* (2016)[1] find ε to be 0.7 *via* simulations and 2.7 *via* theory. These considerations are done for a droplet sized much smaller than a mammalian cell characterized in our work. J.J. Ruz *et. al* (2014)[5] determines the stiffness effect to be

$$(\Omega_{\mathrm{m}})_{\mathrm{stiffnes}} = \left(\frac{1}{\beta_{\mathrm{n}}^4}\right) p_{\mathrm{rec}}(\eta_{\mathrm{rec}}) * \left(\frac{d^2\psi(x_0)}{dx^2}\right)^2 \left(\frac{E_{\mathrm{a}}}{E_{\mathrm{b}}}\right)\left(\frac{V_{\mathrm{a}}}{V_{\mathrm{b}}}\right) \qquad \text{Eq. R2}$$

with V_{a} and V_{b} being the volumes of absorbate and beam, β_n being the eigenvalue coefficients (1.875 for the first shape mode), the relative height difference $\eta_{\mathrm{rec}} = \frac{h_{\mathrm{a}}}{h_{\mathrm{b}}}$, and $p_{\mathrm{rec}}(\eta_{\mathrm{rec}}) = 1.5 + 3\eta_{\mathrm{rec}} + 2\eta_{\mathrm{rec}}^2$ (Eq. R3). The smallest volume of our microcantilevers used is 4000 μm^3, the volume of a rounded HeLa cell having a diameter of ≈ 15 μm is ≈ 1700 μm^3, hence $\left(\frac{V_a}{V_b}\right) \approx 0.42$. With $\eta_{\mathrm{rec}} = 15$, we find $p_{\mathrm{rec}}(\eta_{\mathrm{rec}}) = 496$. These considerations show, that ε will be about 200. For a bigger cell diameter ε might go up to 1000, but not beyond.

There are, however, two assumptions of the above equations R2 and R3, provided by J.J. Ruz *et. al* (2014)[5]. Firstly, a rectangular absorbate is used and, secondly, the absorbate is reasonably small. Whereas the exact shape of the adsorbate will certainly influence the exact result, it cannot account for changing the results by several orders of magnitude. At the same time, although the size of the adsorbate may be relevant, it should not influence the mode shape of the cantilever. In addition, we kindly note that we did not observe any mode shapes of the microcantilever in our FEM-simulations (Figure R4, which we took from the first round of our response to reviewer #1).

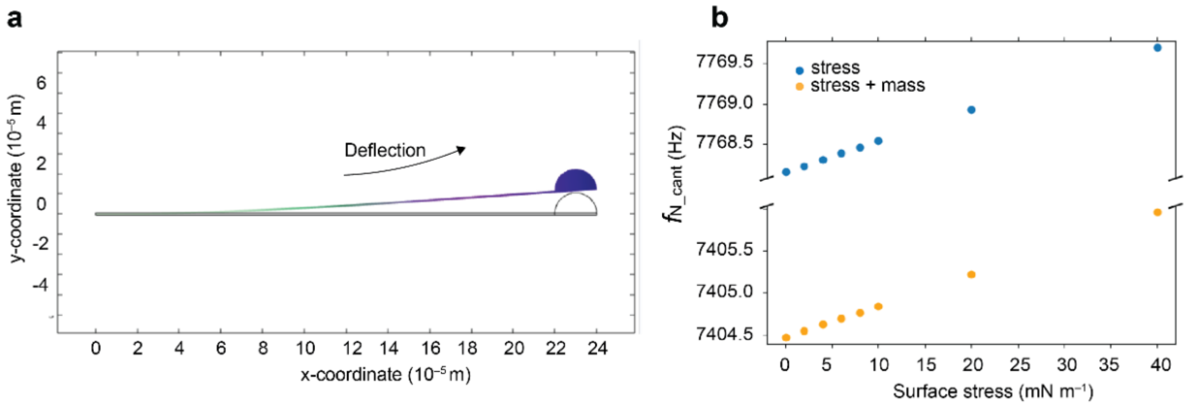

Figure R4 | Finite element simulations showing that the surface stress induced spring constant change and shift of natural resonance frequency of the microcantilever by the adherent cell is negligible compared to the changes caused by the mass added by the adherent cell. a, COMSOL model and mode shape of the microcantilever with attached cell. In black and white is the original, undeflected cantilever and cell geometry. The colorized deflected cantilever geometry shows that the bending of the cantilever predominantly occurs in the region of changing color (i.e., towards the cantilever center) and not at the region of the cell attachment. **B**, Change in natural resonance

frequency of the cantilever as a function of the surface stress applied and the mass of 5.5 ng added by an adhering cell. The cell parameters assumed for this simulation were those of a typical fibroblast in the adherent state having a diameter of ≈ 20 µm and applying a cantilever surface stress increase δs_{cant} of up to 20 mN m^{-1}. Figure has been taken from the first round of our response to reviewer 1.

Conclusion: The stiffness effect of the cell on the natural resonance frequency (eigenfrequency) shift of the microcantilever is negligibly small, also in the theoretical framework provided by Malvar *et al.* (2016).[1] The reason is that although the HeLa cell is relatively large ($\varepsilon \approx 10^3$), the stiffness of the HeLa cell compared to that of the microcantilever is insignificant ($\frac{E_{\mathrm{a}}}{E_{\mathrm{b}}} \approx 10^{-8}$). In addition, in our experiments the HeLa cell is placed at an insensitive position (free end) of the cantilever where no bending occurs ($\kappa_{\mathrm{n}}^2 \approx 10^{-2}$). This observation is also in line with our experiments, which show higher mass readouts for stiffened (crosslinked) cells (Figure 2d and 5e,f), whereas according to Tamayo *et al.* the stiffness effect (an increase in cell stiffness) should lead to a lower mass readout. Furthermore, our FEM simulations also show that the stiffness effect is orders of magnitude too small to have any impact on our measurements or conclusions (Figure 3). Accordingly, we have briefly outlined into our revised manuscript why the stiffness effect of the cell plays a negligible role in our mass measurements (see revised Introduction and Supplementary Note 1). We again thank the reviewer for sharing his/her concerns with us and hope that we could convincingly show that the stiffness effect has no measurable impact on our results.

References

1 Malvar, O. *et al.* Mass and stiffness spectrometry of nanoparticles and whole intact bacteria by multimode nanomechanical resonators. *Nat Commun* **7**, 13452, doi:10.1038/ncomms13452 (2016).

2 Martinez-Martin, D. *et al.* Inertial picobalance reveals fast mass fluctuations in mammalian cells. *Nature* **550**, 500-505, doi:10.1038/nature24288 (2017).

3 Hopcroft, M. A., Nix, W. D. & Kenny, T. W. What is the Young's Modulus of Silicon? *J Microelectromech Syst* **19**, 229-238, doi:10.1109/Jmems.2009.2039697 (2010).

4 Hayashi, K. & Iwata, M. Stiffness of cancer cells measured with an AFM indentation method. *J Mech Behav Biomed Mater* **49**, 105-111, doi:10.1016/j.jmbbm.2015.04.030 (2015).

5 Ruz, J. J., Tamayo, J., Pini, V., Kosaka, P. M. & Calleja, M. Physics of nanomechanical spectrometry of viruses. *Sci Rep* **4**, 6051, doi:10.1038/srep06051 (2014).

REVIEWER COMMENTS

Reviewer #1 (Remarks to the Author):

Referee Report on the second revision of the Manuscript of the paper "Monitoring the mass, eigenfrequency, and quality factor of mammalian cells" by S. Herzog et al., submitted to Nature Communications.

I admit that the authors did a huge job answering the reviewers' comments, but in my turn, I am also "somewhat surprised" that my main question remains unanswered. This is not the cells themselves (i.e. just the circumstance that some "bulbs" are attached onto the still unbent vibrating beam) that can cause the material change of the resonance frequency, but the circumstance that attached cells cause the bending of the cantilever (so that important change of its general shape occur) due to the stress induced by them. Then this bent cantilever may have very different "hydrodynamic function" and, as a result, quite different resonant frequency when dither in water. I fully agree that the effect of the "shapes of cells" is quite small.

To certain extent, rather related questions are considered in the answers of the authors to the third reviewer. However, it should be noted that the theory and experiments reported by Malvar et al. are done in air (at the pressure of 0.1 Tor for experiments described in Nat. Comm. 2016, to be precise); essential modifications are needed to discuss the corresponding situation in liquid. Moreover, it seems that the change of the cantilever shape caused by the local stress related with attached bodies (cells in the case) is not considered: "in this model, we assume that molecules adsorbed on the cantilever negligibly change the eigenmode shape", p. 224104-1 of J. Tamayo et al., Appl. Phys. Lett. 89, 224104 (2006).
Thus unfortunately I do not see the reasons to change my recommendation.

Reviewer #3 (Remarks to the Author):

The authors have addressed my comments. The manuscript might be suitable for publication in Nature Communications.

NCOMMS-23-02495C

Monitoring the mass, eigenfrequency, and quality factor of mammalian cells

Point-by-point response to the comments of reviewer #1

Reviewer: I admit that the authors did a huge job answering the reviewers' comments, but in my turn, I am also "somewhat surprised" that my main question remains unanswered. This is not the cells themselves (i.e. just the circumstance that some "bulbs" are attached onto the still unbent vibrating beam) that can cause the material change of the resonance frequency, but the circumstance that attached cells cause the bending of the cantilever (so that important change of its general shape occur) due to the stress induced by them. Then this bent cantilever may have very different "hydrodynamic function" and, as a result, quite different resonant frequency when dither in water. I fully agree that the effect of the "shapes of cells" is quite small.

To certain extent, rather related questions are considered in the answers of the authors to the third reviewer. However, it should be noted that the theory and experiments reported by Malvar et al. are done in air (at the pressure of 0.1 Tor for experiments described in Nat. Comm. 2016, to be precise); essential modifications are needed to discuss the corresponding situation in liquid. Moreover, it seems that the change of the cantilever shape caused by the local stress related with attached bodies (cells in the case) is not considered: "in this model, we assume that molecules adsorbed on the cantilever negligibly change the eigenmode shape", p. 224104-1 of J. Tamayo et al., Appl. Phys. Lett. 89, 224104 (2006). Thus unfortunately I do not see the reasons to change my recommendation.

Authors: Thank you for acknowledging our effort in addressing the reviewers' comments.

In our first response to reviewer #1, we provided analytical and numerical evidence that the surface stress applied by the cell induces a natural frequency (eigenfrequency) shift of the microcantilever, which can safely be neglected in our experimental setup, as this shift is too small compared to the frequency shift caused by the cell mass and, in addition, has the wrong algebraic sign to explain our experimental results. We were surprised that the reviewer did not engage with this line of reasoning, instead bringing forward the hydrodynamic function of the microcantilever affected by the presence of the cell. Therefore, in our second answer, we discussed the drag force on the microcantilever, which is not being affected by the cell attachment. Yet, with the new formulation of the reviewer's #1 concern above, we understand that the reviewer asks if stress *as a secondary effect* induces a frequency shift of the microcantilever by inducing a change of the mode shape, which would not cause a frequency shift in air, but could potentially change the hydrodynamic function of the cantilever in liquid and therefore change the mass measured in our experimental setup. In the following, we try to shed additional light on the matter from different perspectives.

Firstly, microcantilevers commonly experience static bending due to internal stress [1, 2], particularly in a liquid environment. This is a known concern for applications of static force spectroscopy but to our best knowledge has hardly been of concern for dynamic applications and hence has not been considered to hypothetically change the hydrodynamic function of the cantilever. However, we performed additional experiments to gauge the possible effect of surface stress induced by a cell adhering to a microcantilever (Fig. R1). To this end, we

recorded the thermal noise spectrum of a 120 μm long microcantilever without a cell, to find its Q factor and natural resonance frequency $f_{\mathrm{N\_cant}}$. We then positioned a second laser (additional to the readout laser) 15 μm from the free end of the microcantilever and applied a static laser power of 1 mW (Fig. R1a). This photothermal heating and the associated bimetal effect of the gold-coated cantilever induced mechanical stress that deflected the microcantilever by ≈ 0.5 nm (Fig. R1b). The photothermally induced stress was designed to mimic ‘a massless cell’ that causes stress in the plane on the microcantilever without loading any mass onto the cantilever. We again measured Q factor and $f_{\mathrm{N\_cant}}$ of the microcantilever and observed a change of ≈ 0.04% in the Q factor and 0.08% in $f_{\mathrm{N\_cant}}$ (Fig. R1c), which would correspond to a mass change of ≈ 0.05 ng. This apparent increase in mass is 10 – 100 times smaller compared to the inertial cell mass of ≈ 3.1 ng (Fig. 1). Considering that the Q factor virtually does not change and that the slight drop in $f_{\mathrm{N\_cant}}$ could also be explained by the photothermal heating (*e.g.* expansion of the cantilever), this finding demonstrates that the thermally induced stress does not affect the hydrodynamic function of the microcantilever.

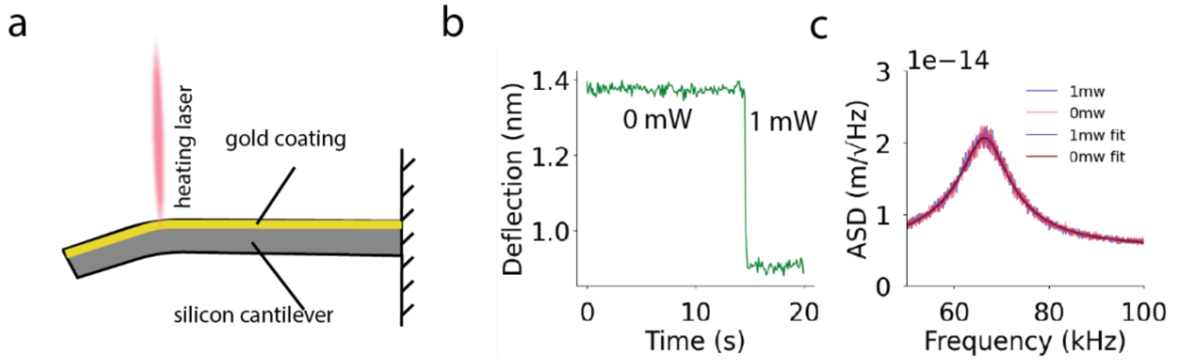

Figure R1. Photothermal deflection of a microcantilever induced by a static laser. a, Illustration of a static laser (1 mW, 840 nm) positioned at 15 μm distance from the free end of a 120 μm long microcantilever. The photothermal heating induces the gold-coated microcantilever to deflect. The deflection of the cantilever is read out by a second laser positioned at the free end of the cantilever (not shown). **b**, Deflection of the microcantilever exposed to the static laser described in **a**. Upon switching the static laser ‘on’ the cantilever deflects ≈ 0.5 nm. **c**, Natural resonance frequency $f_{\mathrm{N\_cant}}$ and Q factor of the microcantilever not exposed (0 mW) and exposed (1 mW) to the static laser. Being, not exposed to the static laser the microcantilever shows a natural eigenfrequency $f_{\mathrm{N\_cant}}$ of 66834 ± 25 Hz and a Q factor of 6.853 ± 0.07. Exposed to the static laser the microcantilever shows a natural eigenfrequency $f_{\mathrm{N\_cant}}$ of 66785 ± 25 Hz and a Q factor of 6.856 ± 0.03. Experiments were conducted in aqueous solution at room temperature.

Secondly, as outlined in our first response to reviewer #1, we estimate that the change in microcantilever stress caused by the attached cell to be $\delta s_{\mathrm{cant}} \leq -1$ mN m^{-1}. To set an upper limit of the deflection induced by the cell stress, we use the formula: $\delta z = \frac{4l^2 \delta s_{\mathrm{cant}}(1-\nu)}{Et^2}$ [3], where t and l are the thickness and length of the microcantilever, respectively. E and ν are Young’s modulus and Poisson ratio, respectively. Using $E = 200$ GPa, $\nu = 0.35$, $t = 600$ nm, and $l = 100$ μm, we get $\delta z = 0.4$ nm. This deflection is in agreement with our experimental experience and puts the cell-induced deflection at the same order of magnitude as the photothermally induced static deflection. Therefore, we conclude that our experimental setup (Fig. R1) is a good way to assess possible cell stress-induced effects on the microcantilever.

We further set out to explore the theoretical possibility of a changed mode shape, which may be caused by a cell that locally stiffens the microcantilever. To approximate this effect we consider the homogeneous Euler-Bernoulli beam equation $\frac{d^2}{dx^2}\left(EI\frac{d^2}{dx^2}z\right) = 0$. The solution of this equation gives rise to the mode shapes of the microcantilever. Thereby, the product of

Young's modulus and the inertial area moment, EI, is called flexural rigidity, whereas z(x) is the deflection of the microcantilever beam. The flexural rigidity can be written as $EI = wED$, with w being the width and D the bending modulus per unit width of the microcantilever. If we consider the cell in an effective model of a microcantilever beam (Fig. R2), which is composed of two different components, we can write $D = E_1^2 t_1^4 + E_2^2 t_2^4 + 2E_1 E_2 t_1 t_2 (2t_1^2 + 2t_2^2 + 3t_1 t_2)/(12(E_1 t_1 + E_2 t_2))$ [4], with t_i being the thickness of the respective layer (cantilever and cell). Here, instead of working with the stress applied by the cell, we concentrate on the rigidity of the cell, which is the result of the cell stress (as explained by the tensegrity model [5]). Comparing the bending modulus D without and with cell ($E_1 = 200$ GPa, $E_2 = 1\ k$Pa, $t_1 = 600\ nm, t_2 = 20\ um$) we find that D differs by 0.01%, which is two orders of magnitude smaller than the shift of the natural frequency $\Delta f_{\mathrm{N\_cant}}$ of the microcantilever detected in our experiment. Therefore, we can conclude that although the attached cell makes the cantilever effectively much thicker (factor 20), the eight orders of magnitude between the stiffness of silicon ($E_1 = 200$ GPa) and cell ($E_2 = 1$ kPa) makes this effect negligible.

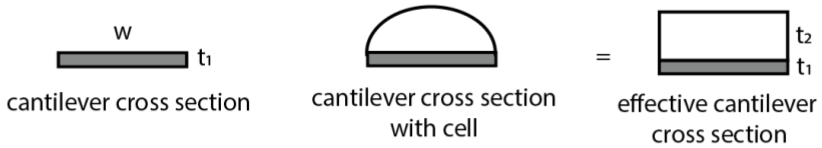

Figure R2. Schematic to calculate the change in cantilever bending rigidity. The microcantilever without cell has a width w and thickness t_1. For calculations of the bending rigidity of the microcantilever with an attached cell, we assume the cantilever being coated by a film of thickness t_2.

For a more numerical argument of the former point, we conducted finite element method (FEM) simulations of the mode shape of the microcantilever. Hereby, we considered the first mode (which is the exclusive basis for the experiments presented in our Manuscript) and the second mode of the microcantilever as a comparison (Fig. R3). For the simulations, the cell was assumed to be a hemisphere with diameter of 20 µm, residing within the last 20% of the free cantilever end. For the simulated cell stiffnesses ranging from 1 kPa (realistic for our mammalian cells) to 1 GPa (for a dehydrated bacterium with cell wall), the first mode shape remains unaffected. In contrast, the second mode shape is already affected by a cell stiffness of 1 kPa. We find the reason in our answer to reviewer #3: *the minimally increased bending modulus D can take effect only when the cantilever is experiencing an appreciable curvature (bending) in the region of increased bending modulus*. In the first mode, this bending is negligible at the free end of the microcantilever. In the light of the above theoretical considerations, we do agree that, in general, an adsorbate could change the bending shape and thus the hydrodynamic function of the microcantilever.

After several simulations, theoretical considerations, and experimental controls, we find no indication that cellular stress plays a role for our experiments with living mamalian cells that are eight magnitudes softer compared to the microcantilever, in experiments where cells are placed at the free end of the cantilever, and in experiments where we measure the first mode of the microcantilever. We once again thank the reviewer for the tenacity helping us to get a greater clarity on a topic that we have only treated intuitively. We have revised our manuscript accordingly (**see new Supplementary Note 2**).

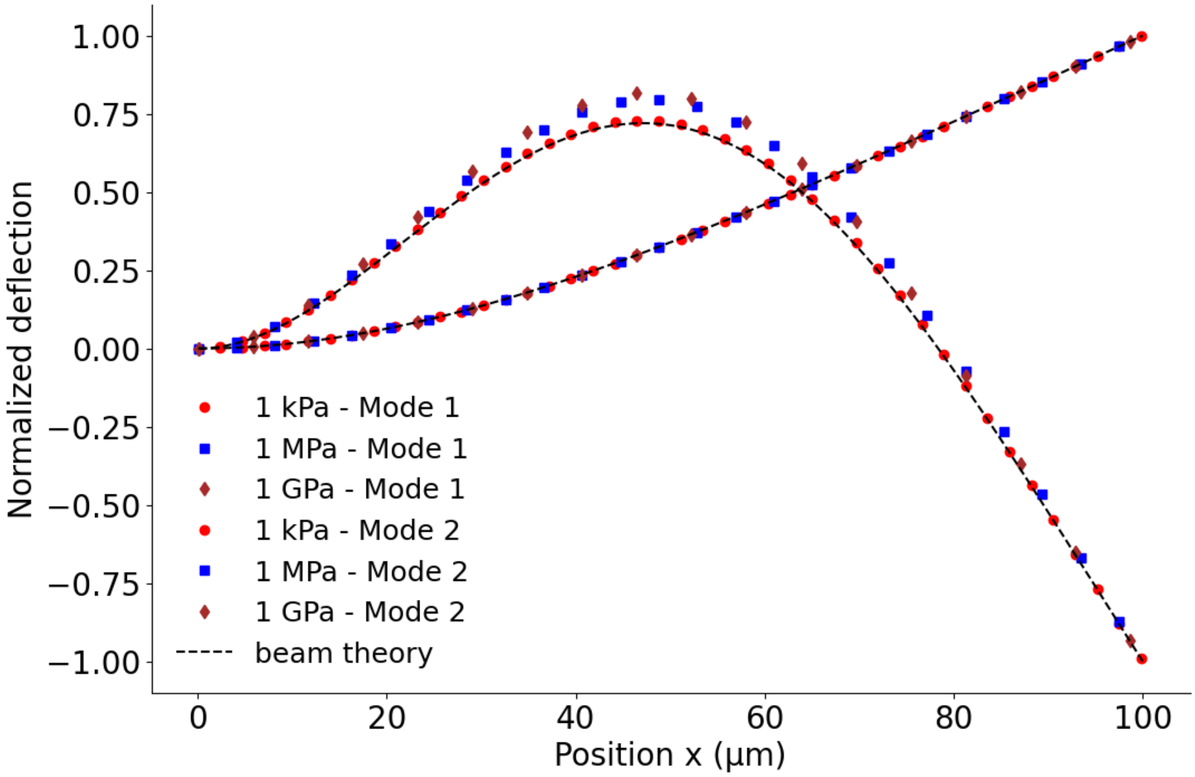

Figure R3. The shape of the first mode stays unaffected by mechanical stiffening of the last 20% of the cantilever length. Shapes of the first and second mode of a 100 µm long microcantilever beam with cells of varying stiffness attached to it, extracted by finite element method simulations. The cell is placed in the last 20% (80 – 100 µm) of the cantilever.

References

1. Huff, M. Residual Stresses in Deposited Thin-Film Material Layers for Micro- and Nano-Systems Manufacturing. Micromachines 13, 2084 (2022). https://doi.org/10.3390/mi13122084

2. Edwards, D. T. & Perkins, T. T. Optimizing force spectroscopy by modifying commercial cantilevers: Improved stability, precision, and temporal resolution. J. Struct. Biol. 197, 13-25 (2017). https://doi.org/10.1016/j.jsb.2016.01.009

3. Miyatani, T. & Fujihira, M. Calibration of surface stress measurements with atomic force microscopy. J. Appl. Phys. 81, 7099–7115 (1997). https://doi.org/10.1063/1.365306

4. Li, X., Shih, W., Aksay, I. A. & Shih, W.-H. Electromechanical Behavior of PZT–Brass Unimorphs. J. Am. Ceram. Soc. 82, 1733–1740 (1999). https://doi.org/10.1111/j.1151-2916.1999.tb01993.x

5. Ingber, D. E. Tensegrity I. Cell structure and hierarchical systems biology. J. Cell Sci. 116 (7), 1157–1173, (2003). https://doi.org/10.1242/jcs.00359

NCOMMS-23-02495C

Monitoring the mass, eigenfrequency, and quality factor of mammalian cells

Point-by-point response to the comments of reviewer #3

Reviewer: The authors have addressed my comments. The manuscript might be suitable for publication in Nature Communications.

Authors: Thank you. Thank you also for your constructive comments made in the previous round and which have guided us to improve our work.

REVIEWERS' COMMENTS

Reviewer #1 (Remarks to the Author):

I can recommend acceptance now. This is a time for the paper to be judged by the whole research community of AFM specialists and not by a few reviewers.

Monitoring the mass, eigenfrequency, and quality factor of mammalian cells

Point-by-point response to the comments of reviewer #1

Reviewer: I can recommend acceptance now. This is a time for the paper to be judged by the whole research community of AFM specialists and not by a few reviewers..

Authors: Thank you for acknowledging our effort in addressing the reviewers' comments. Thank you also for your previous comments, which guided us to revise and improve our manuscript.
